# Supplementary material for: NMDAR mediated dynamic changes in m6A inversely correlates with neuronal translation
Source: Sci Rep. 2022 Jul 5;12:11317. doi: 10.1038/s41598-022-14798-3 (PMC9256623; doi:10.1038/s41598-022-14798-3)
Supplement: Supplementary file 1 — Supplementary Information. [file 41598_2022_14798_MOESM1_ESM.docx]

**Supplementary Figure 1:**

**Supplementary Figure 1: NMDAR stimulation leads to increase in m^6^A levels on RNA which correlates with the global translation inhibition response**

1. Representative immunoblots showing p-eEF2 and eEF2 levels in DIV15 cultured cortical neurons treated with 20 µm NMDA for 1, 5 and 20 minutes and loading control indicated by Tuj1.
2. Quantification of p-eEF2 levels normalized to Tuj1 in DIV15 cultured cortical neurons treated with 20 µm NMDA for 1, 5 and 20 minutes. Data represents mean +/- SEM, n =4 from independent neuronal cultures, One-way ANOVA (p=0.0006) followed by Tukey’s multiple comparison test.
3. Quantitative PCR to access the DNA contamination in RNA isolated from basal condition for mRNA candidates PSD95, PTEN and Actin plotted data points are the Ct values.

**Supplementary Figure 2:**

**Supplementary Figure 2: NMDA induces changes in nuclear and cytosolic levels of m^6^A demethylase FTO**

1. Schematic depicting procedure used for quantification of cyto-dendritic FTO levels.
2. Schematic depicting procedure used for quantification of nuclear FTO levels.

**Supplementary Figure 3:**

**Supplementary Figure 3: NMDAR mediated increase in m^6^A levels is accompanied with shift of m^6^A marked RNA from polysome to non-polysome fractions.**

1. Quantification of 18S rRNA by qPCR method and line graph indicate distribution across the polysome pools

**Raw data:**

**Figure 1:** **NMDAR stimulation leads to increase in m6A levels on RNA which correlates with the global translation inhibition response**

**D:**

**Dot Blot**

**
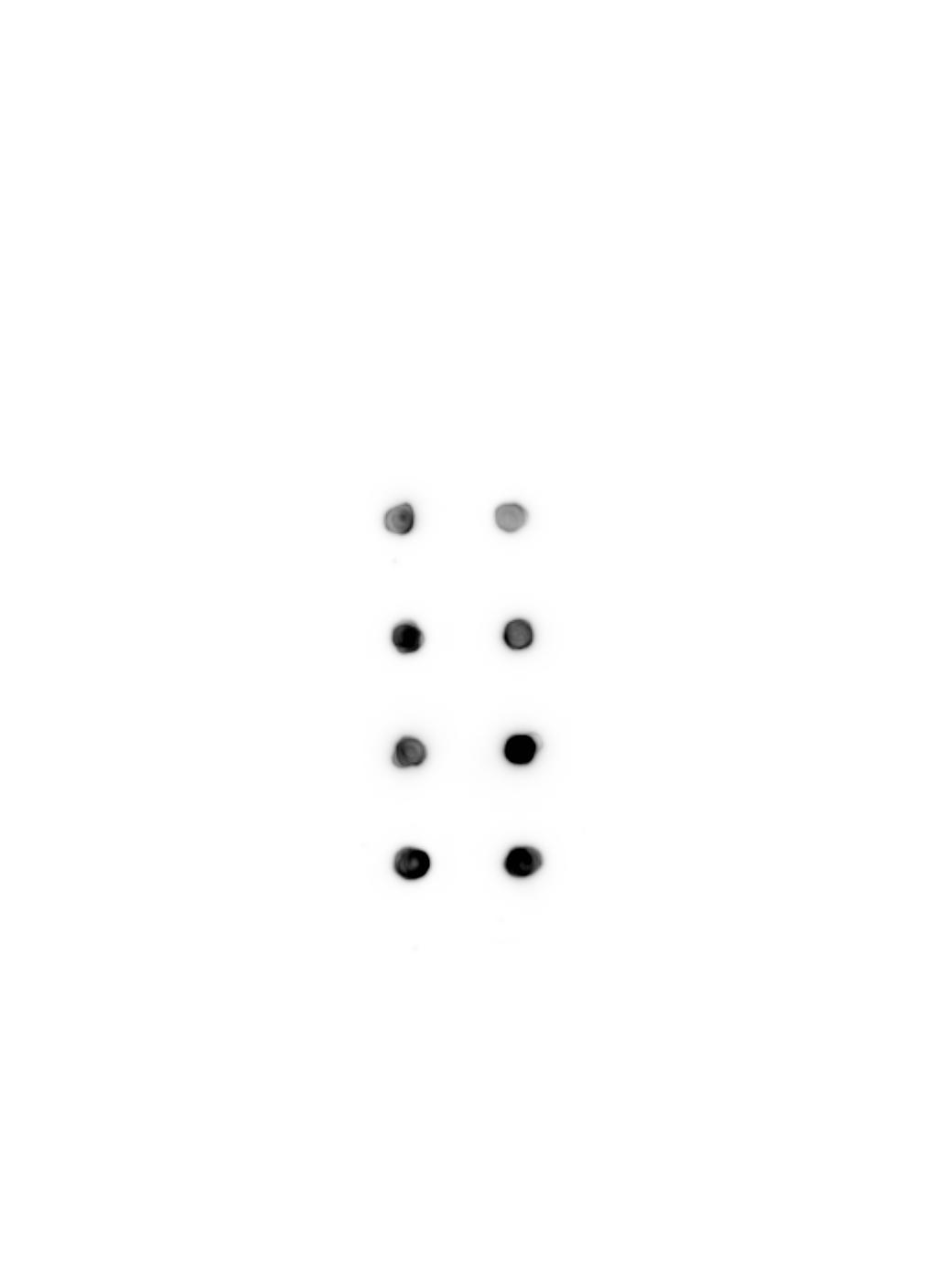
**

**Methylene blue**

**
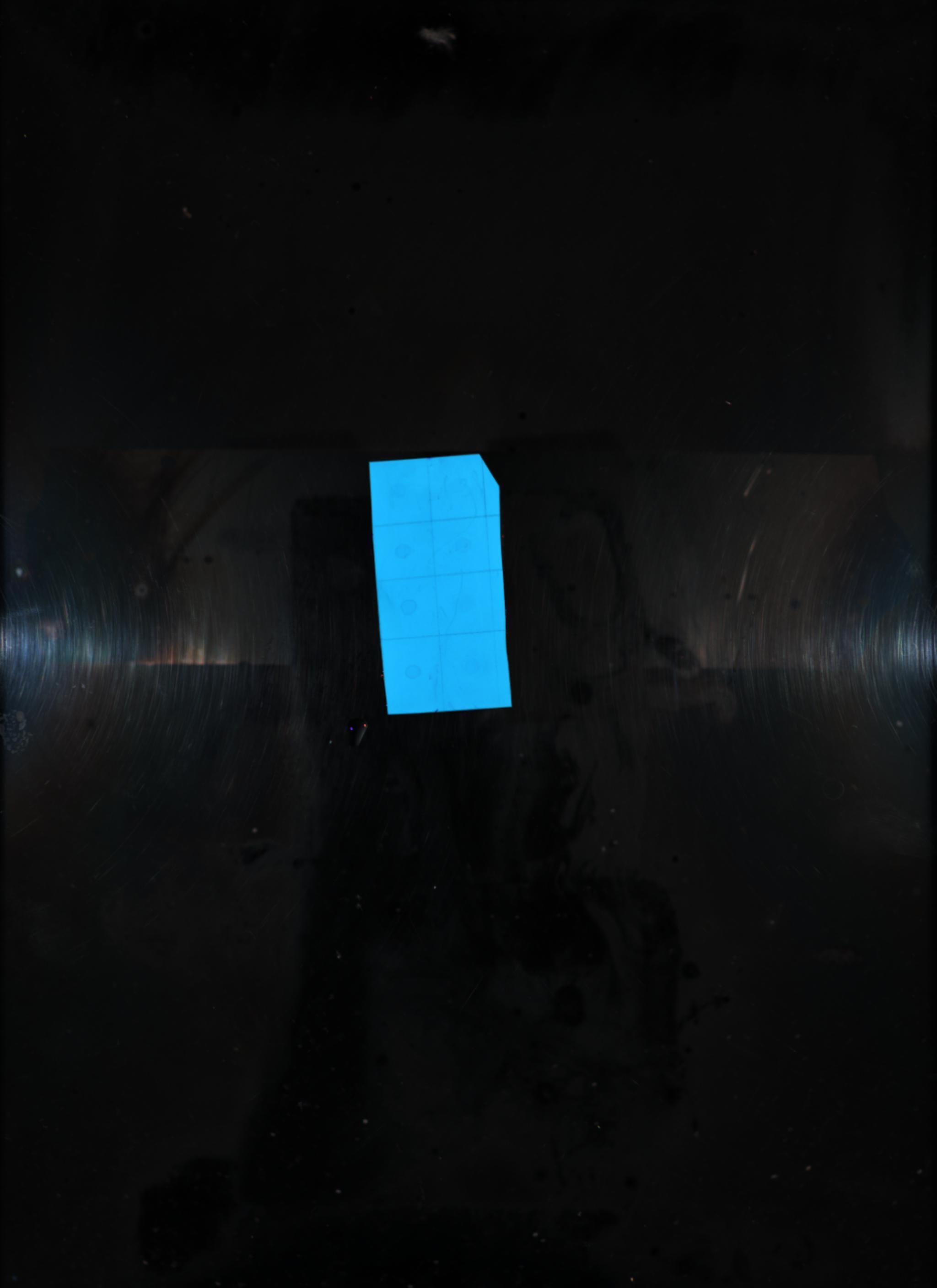
**

**I:**

**Dot Blot**

**
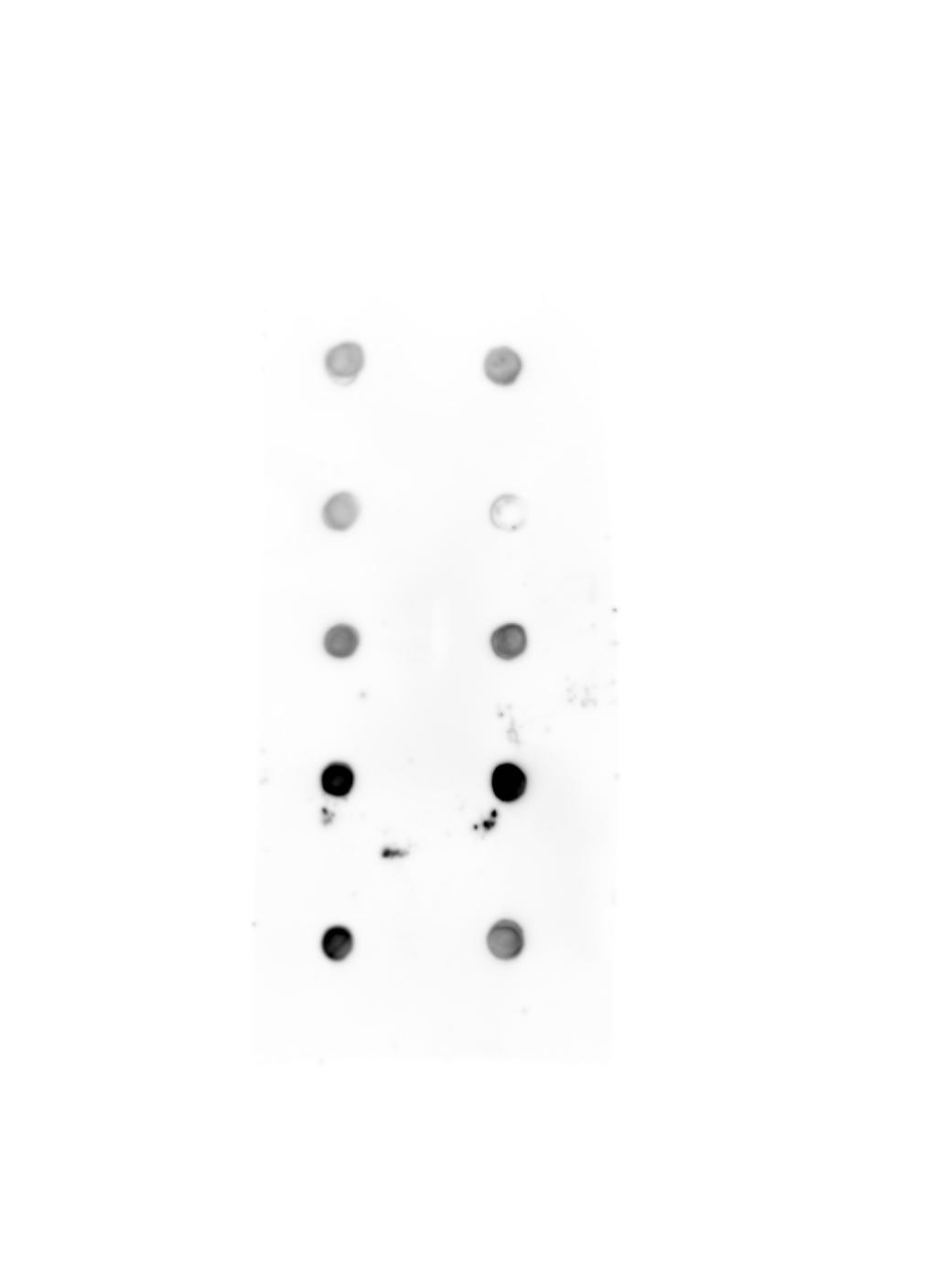
**

**Methylene blue**

**
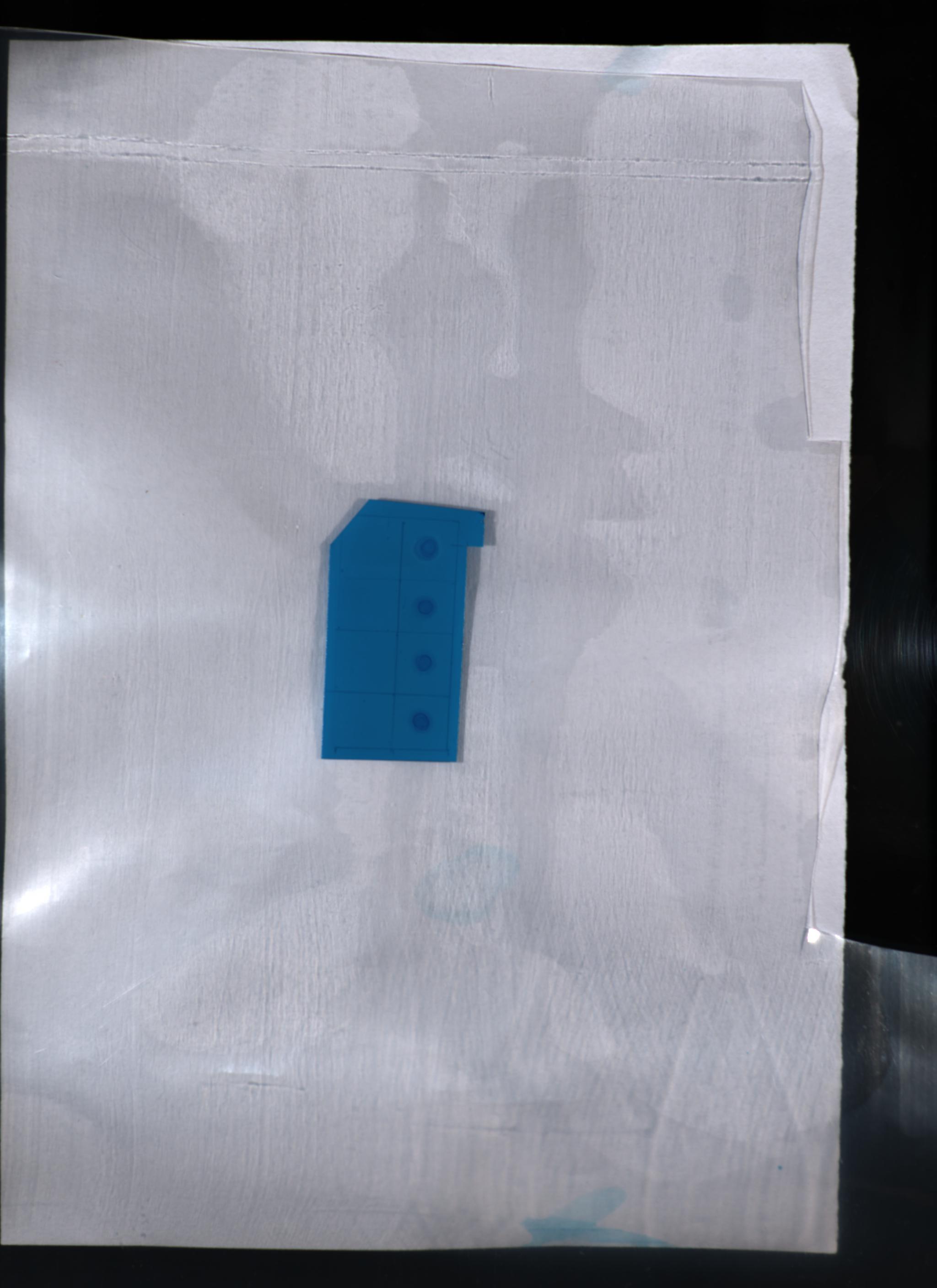
**

**H:**

**PSD95 Blot**

**
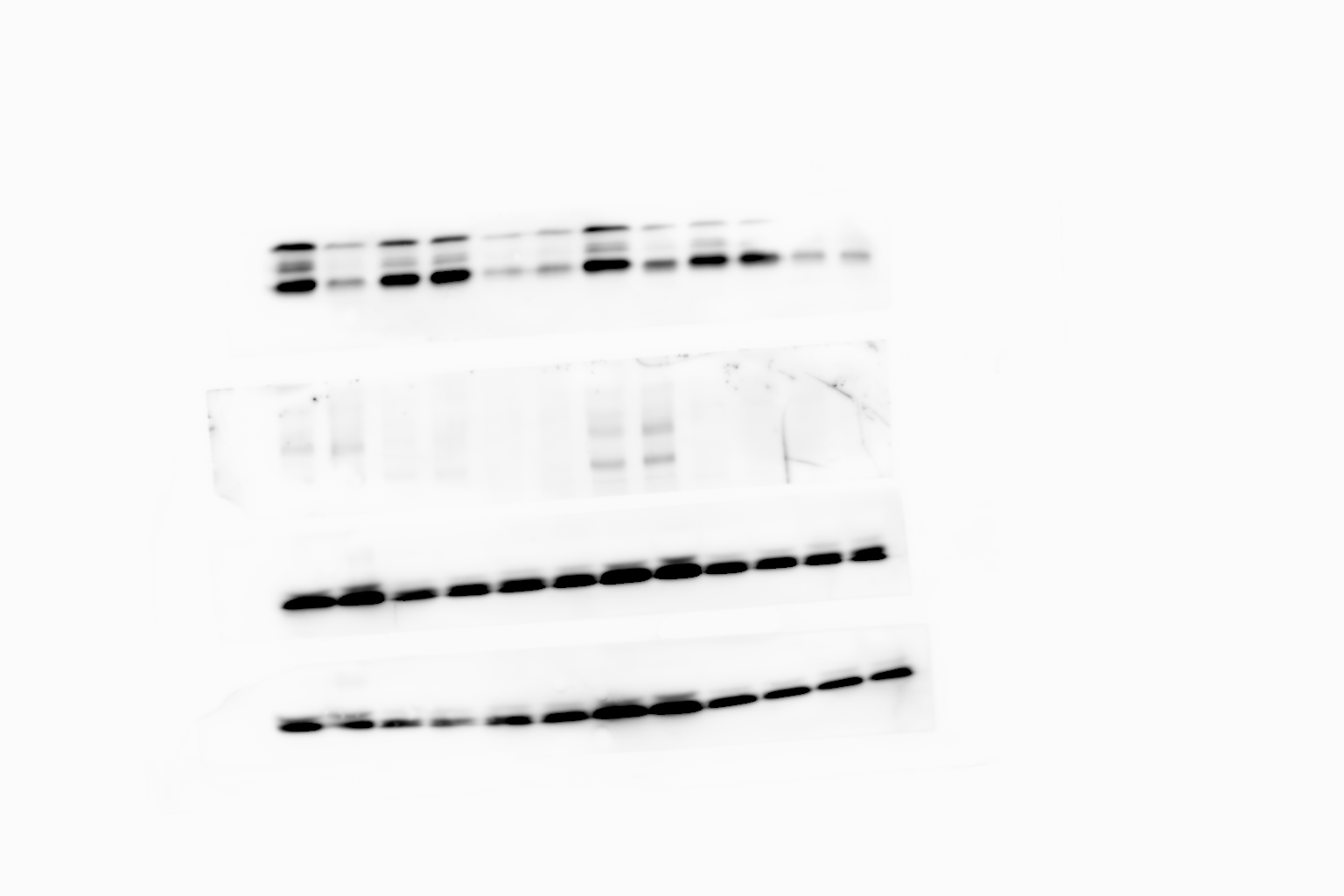
**

**Tubulin:**

**
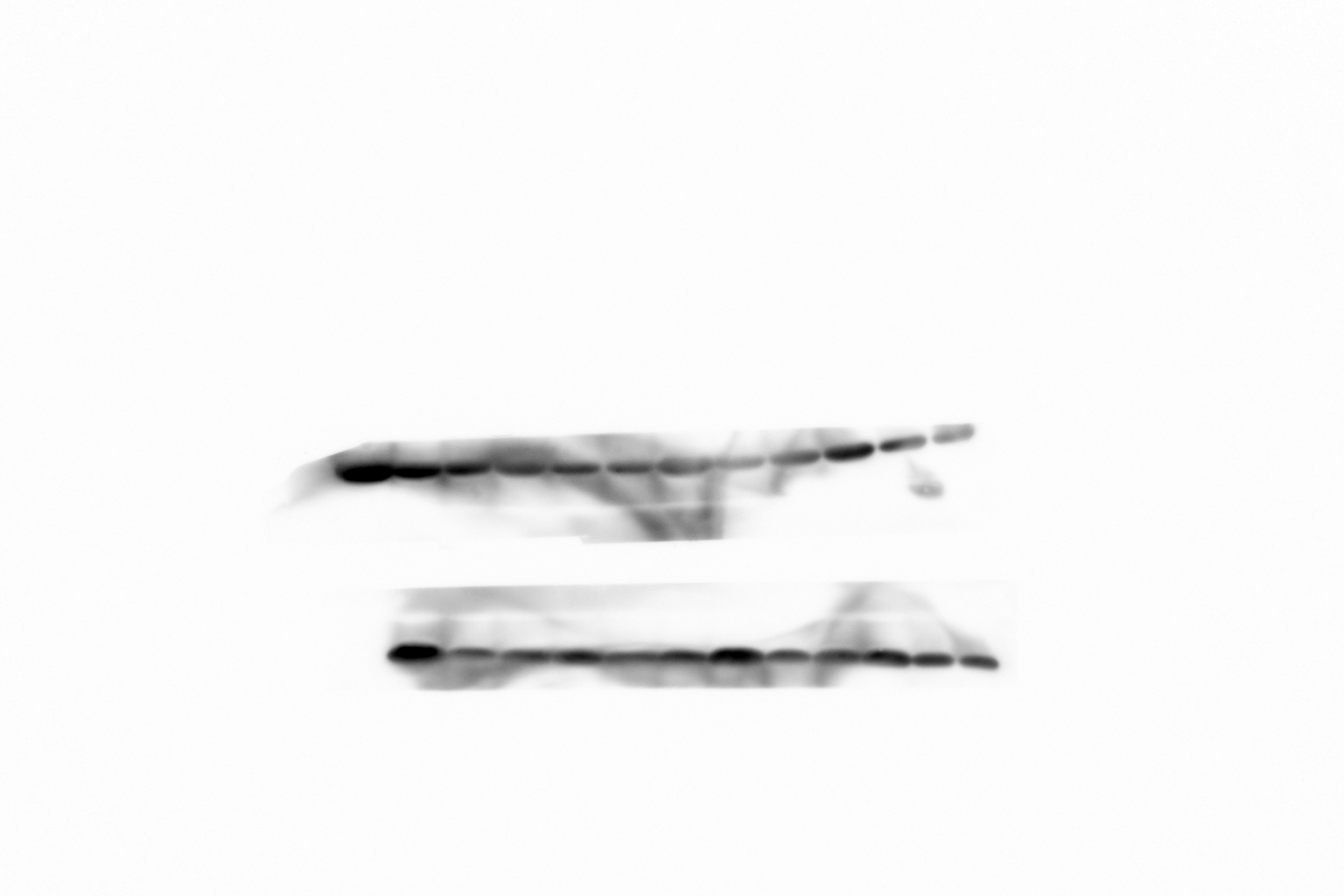
**

**Supplementary Figure 1a:**

**Set1: peEF2:**

Loading order for phospho blots - Ladder, untreated, gap, nifi, n1', nifi+n1', n5', nifi+n5', n20', nifi+n20'

**
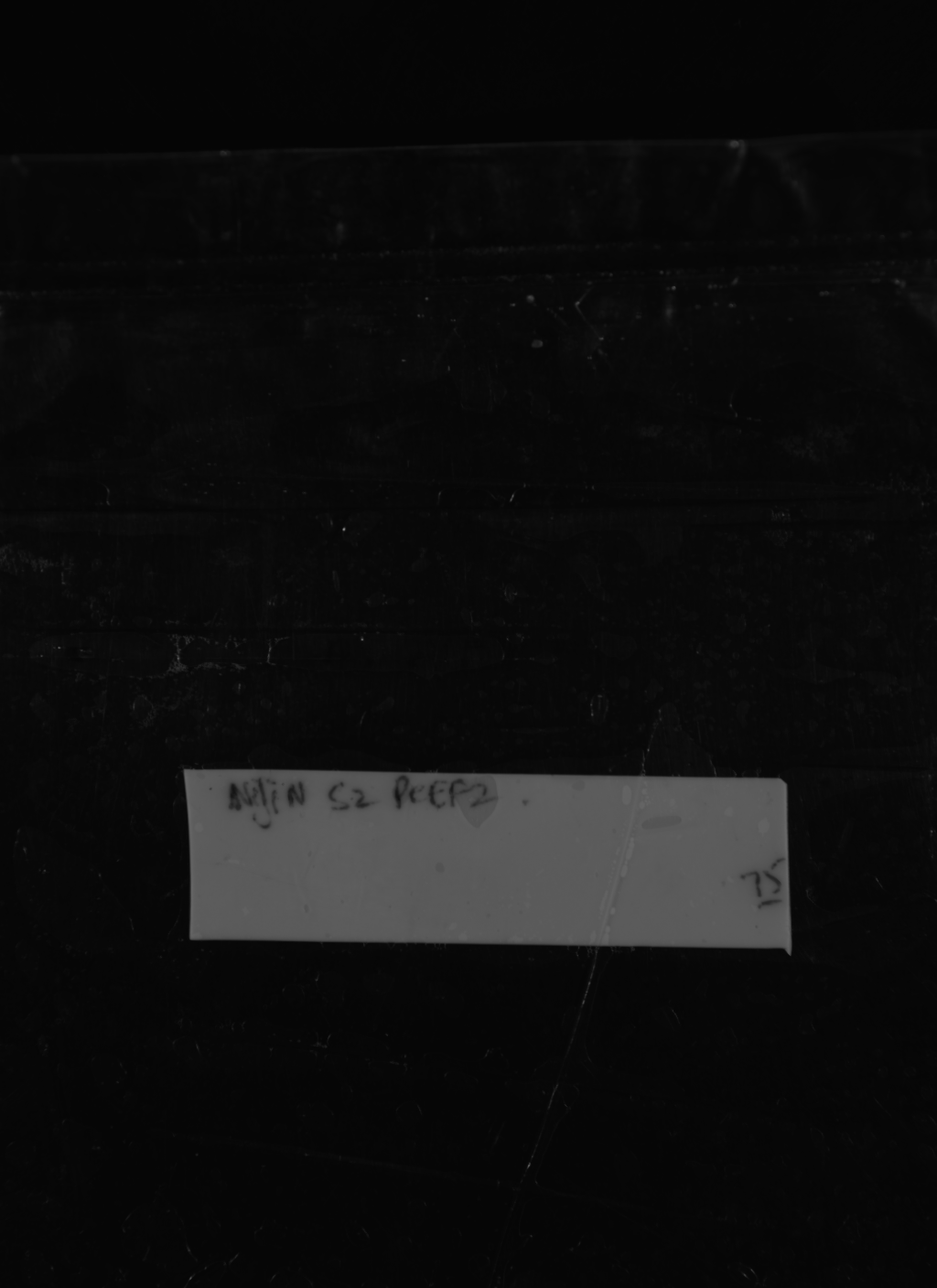

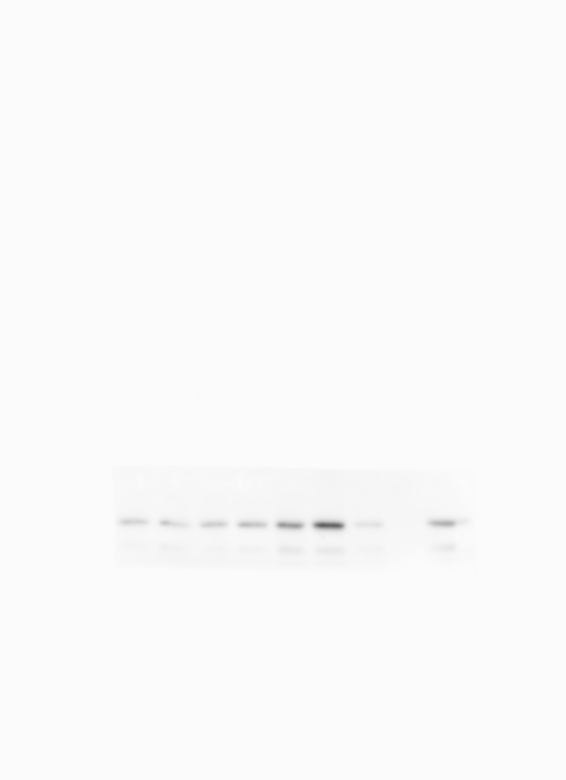
**

**Total eEF2:**

Loading order for total blots - Ladder, untreated, nifi, n1', nifi+n1', n5', nifi+n5', n20', nifi+n20'

**
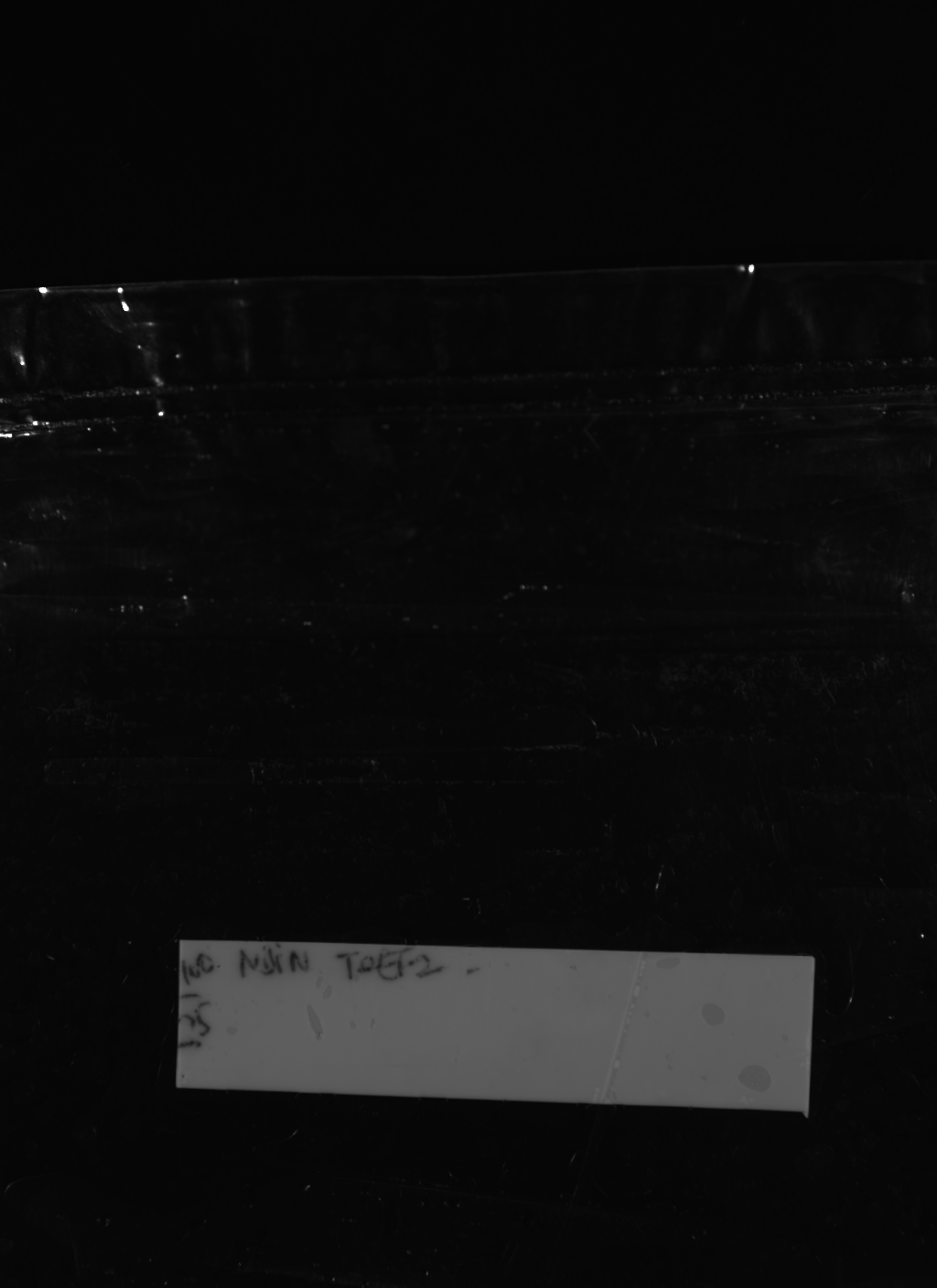
** **
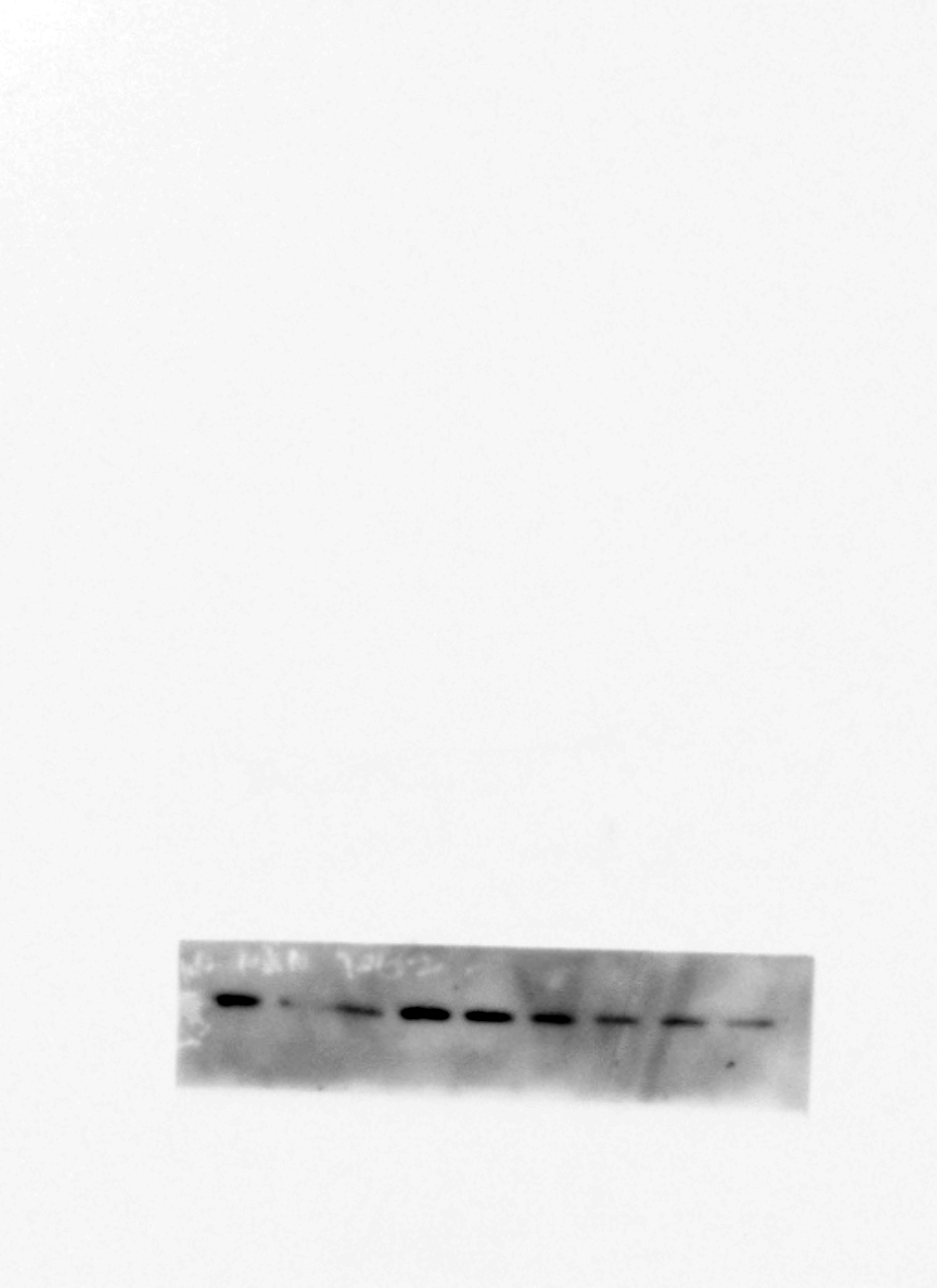
**

**Tuj1:** Tuj1 with marker -  last 2 blots in the image (Tuj1 for p-eEf2 and eEF2 are in the same image)


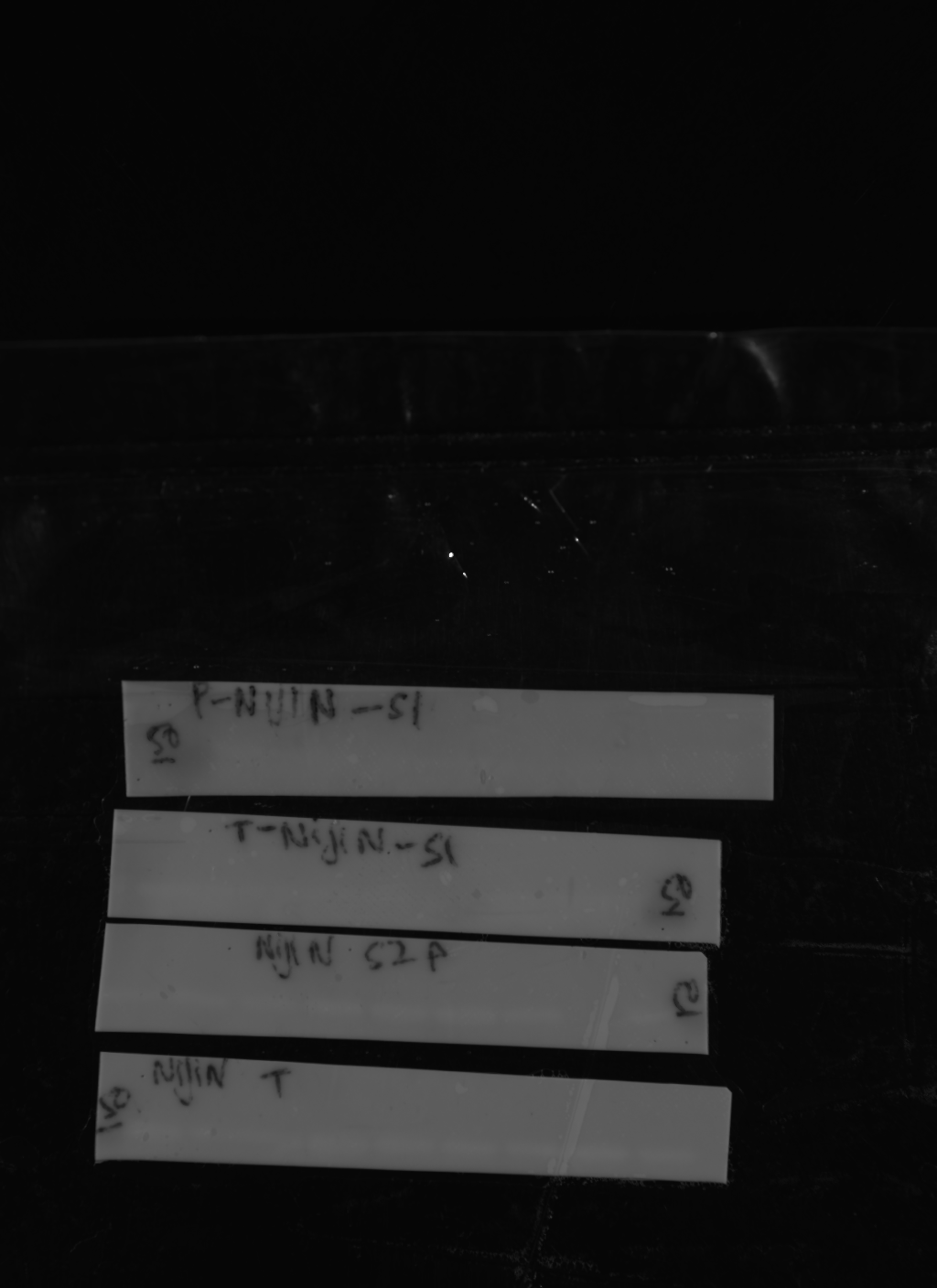

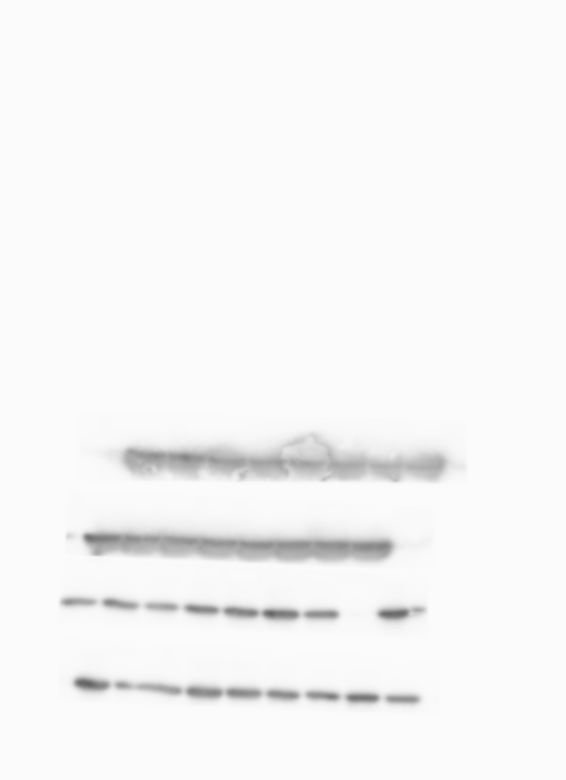


**Set2:**

**peEF2**

Loading order for phospho S1- Ladder, unt, nifi, n1', nifi+n1', n5', nifi+n5', n20', nifi+n20'

**
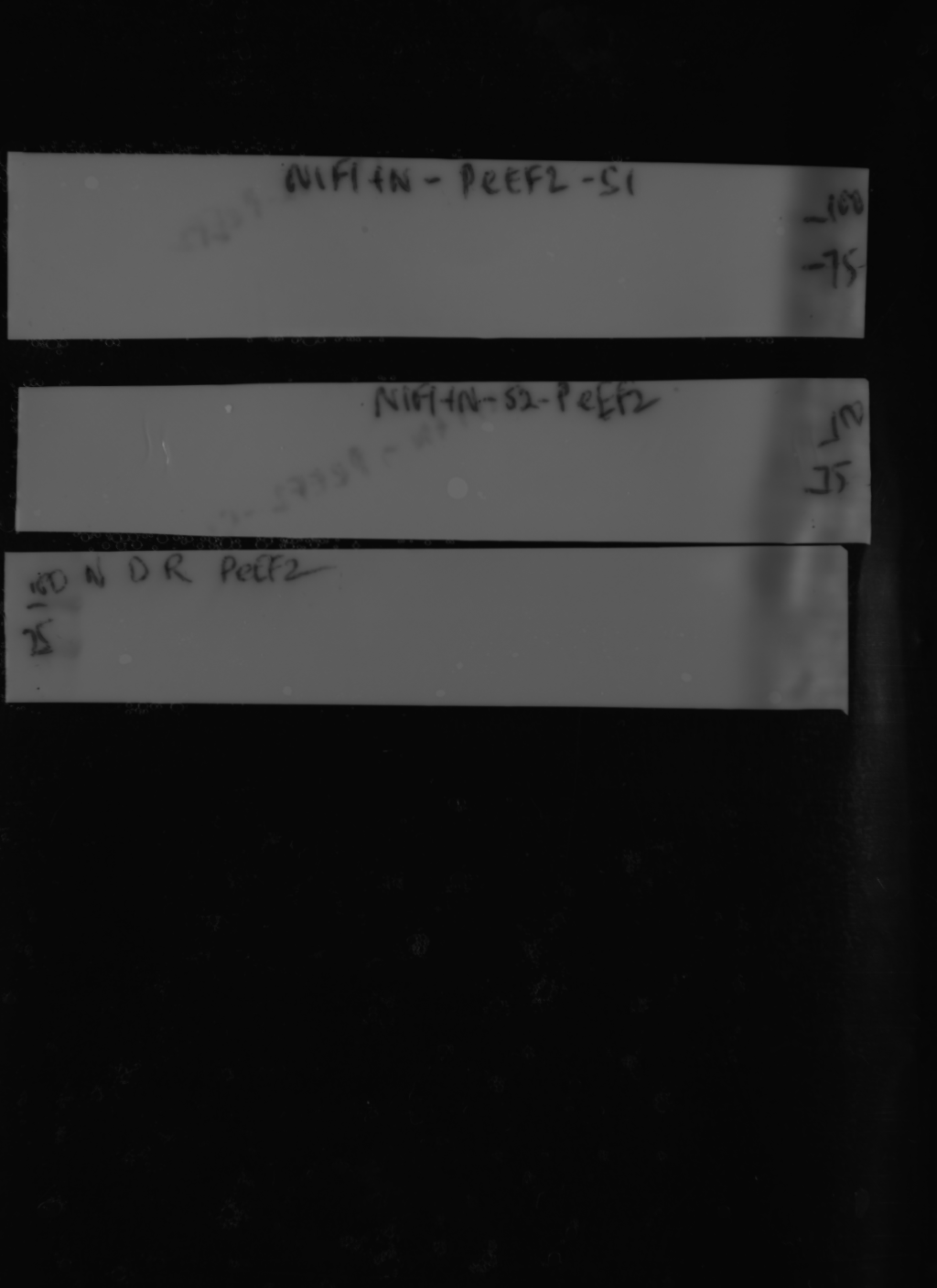

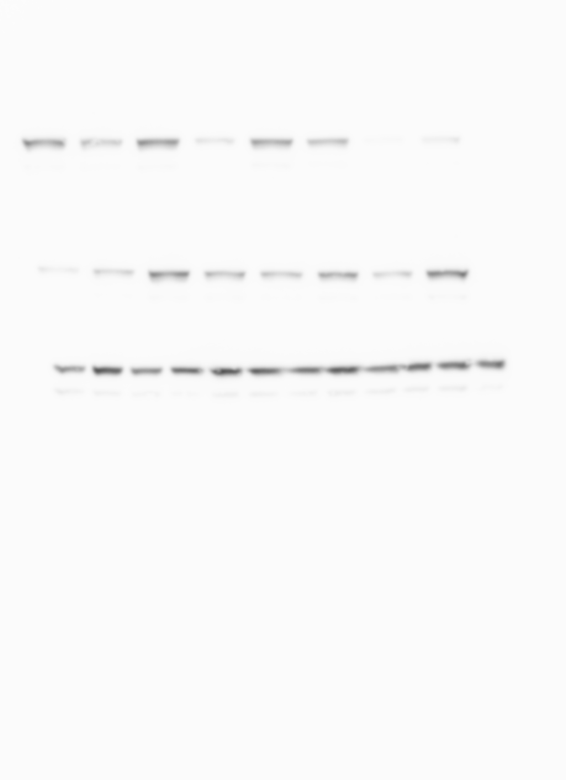
**

Loading order for total S1 - Ladder, unt, nifi, n1', nifi+n1', n5', nifi+n5', n20', nifi+n20'

**Total eEF2**

**
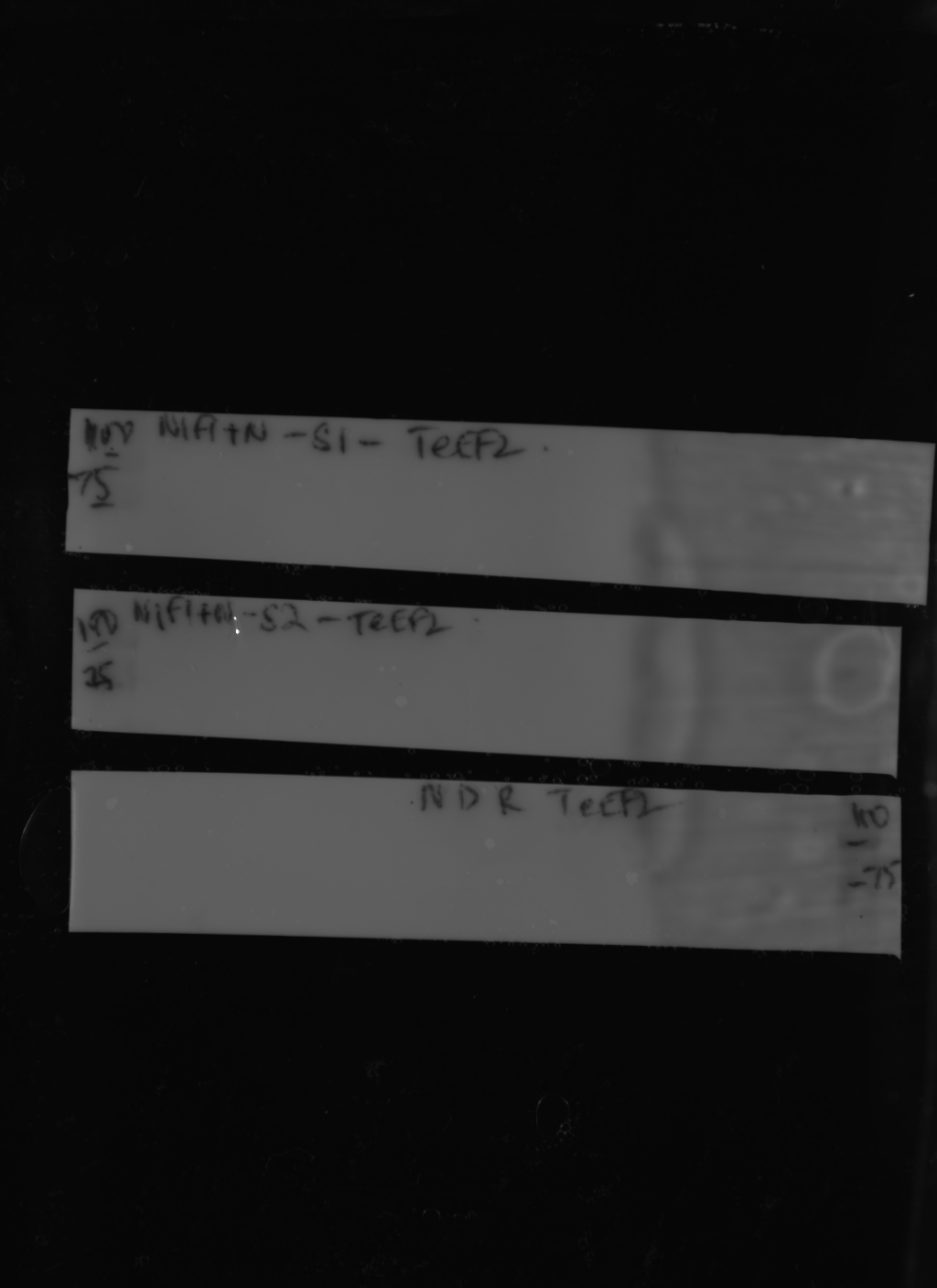
** **
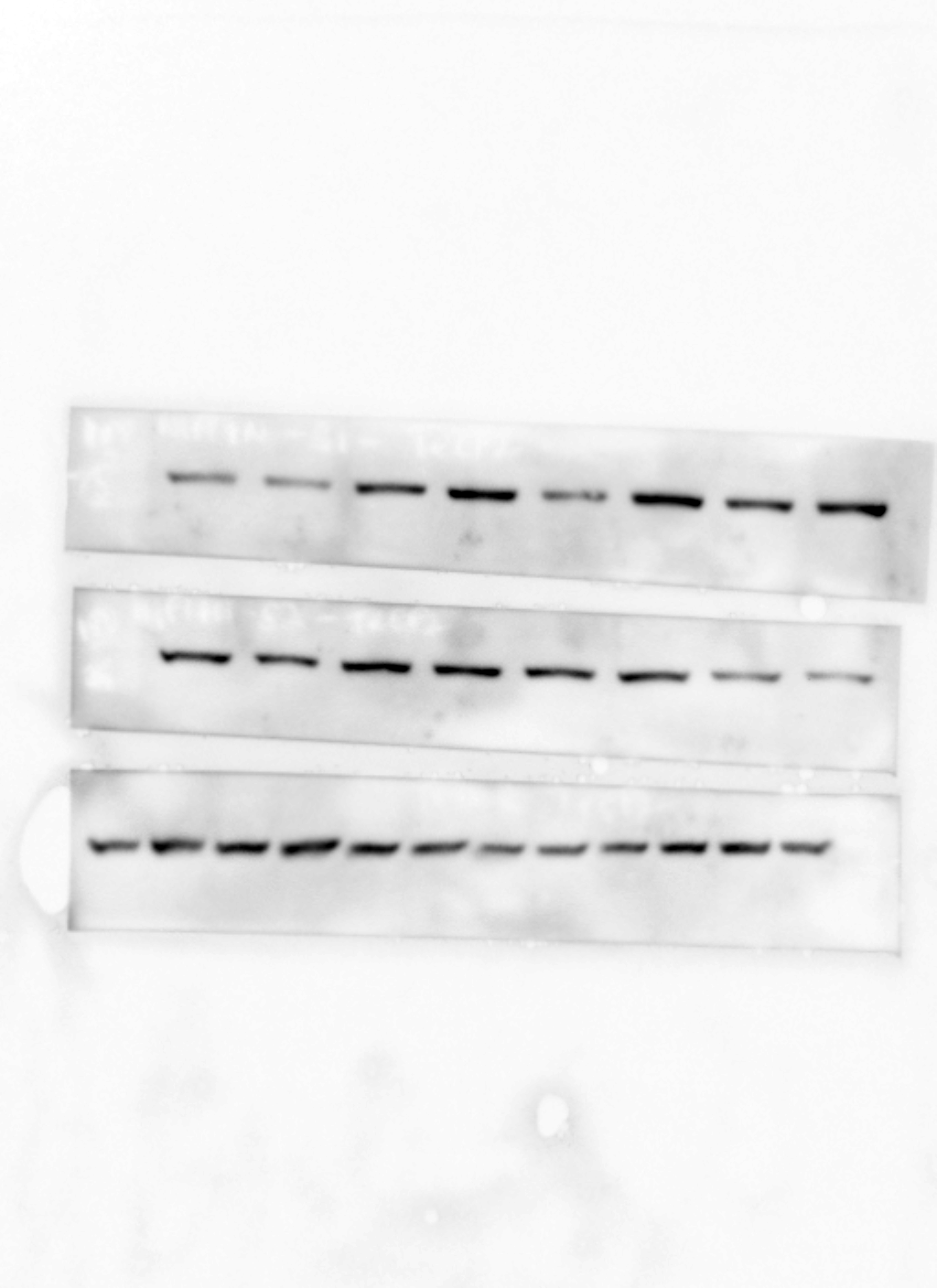
**

**Tuj1:**

Loading order for Tuj1 First two blots - Ladder, unt, nifi, n1', nifi+n1', n5', nifi+n5', n20', nifi+n20'

**
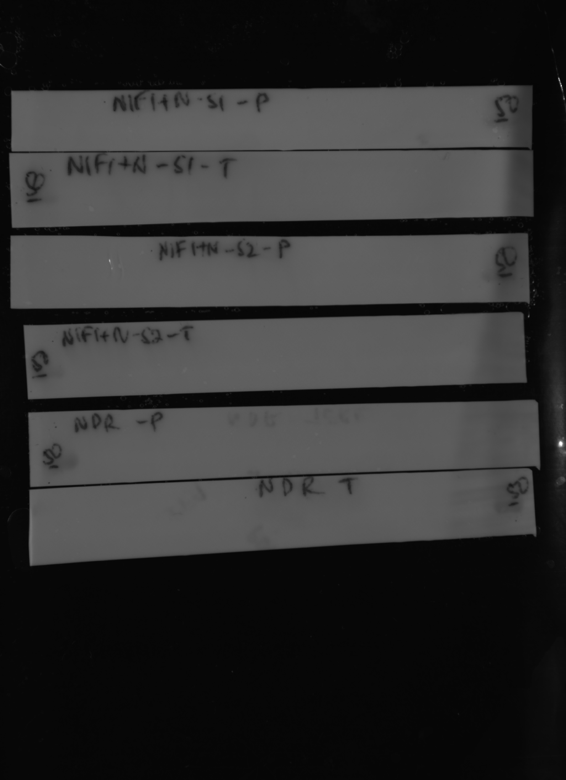

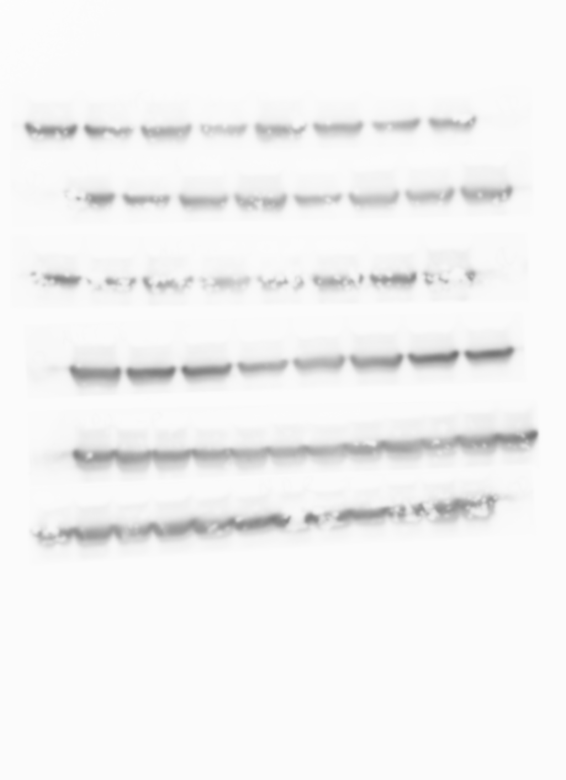
**

**Set3: NMDA Samples are run along with Thapsigargin- for NMDA quantification we have used Set1 samples.**

**Total eEF2:**

Loading order for total eEF2 (set1) - ladder, test sample (it was just an extra sample i had loaded), n1', n5', n20', unt, tg 5', tg 20', tg+n1', tg+n20'

**
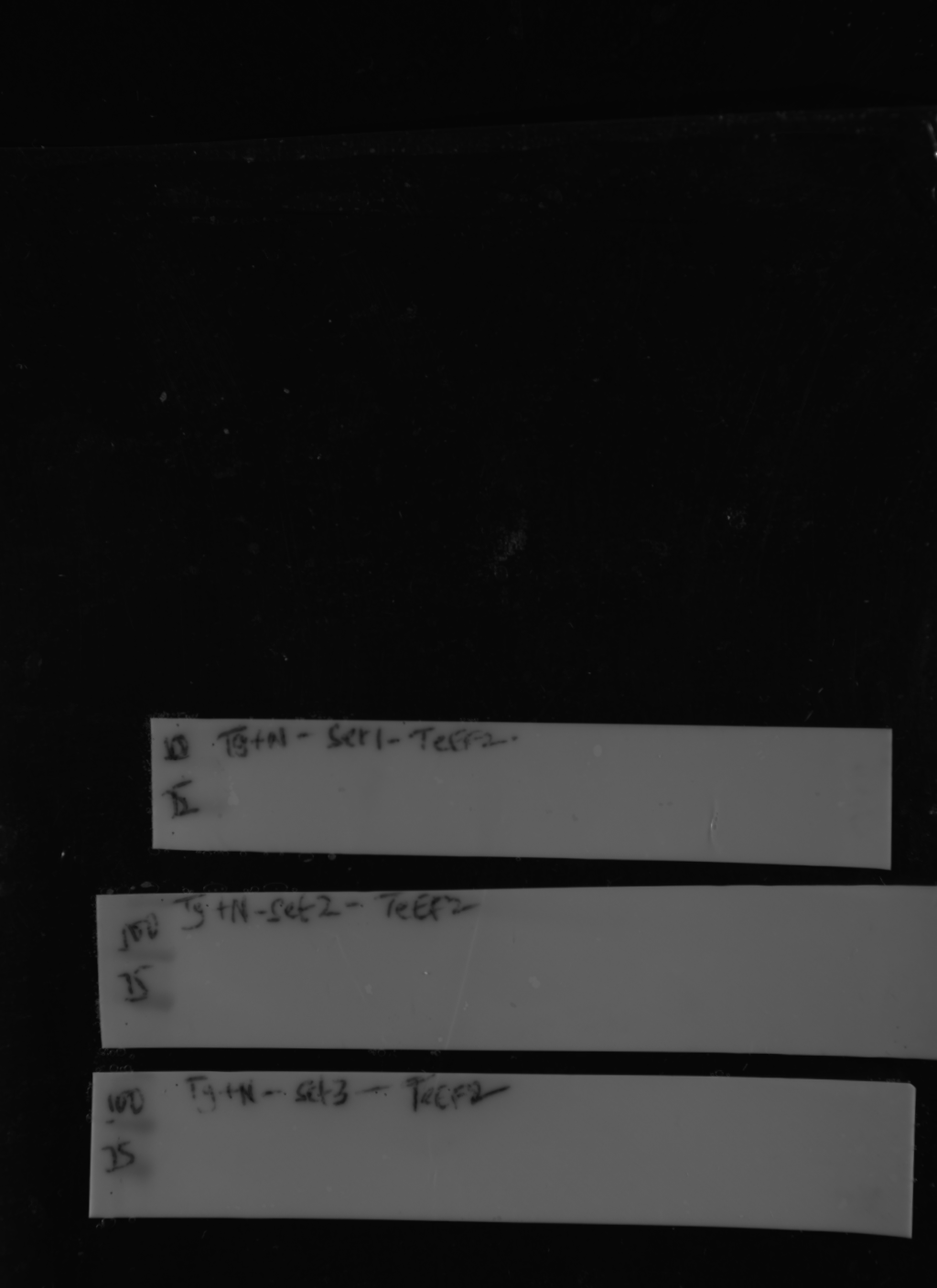
** **
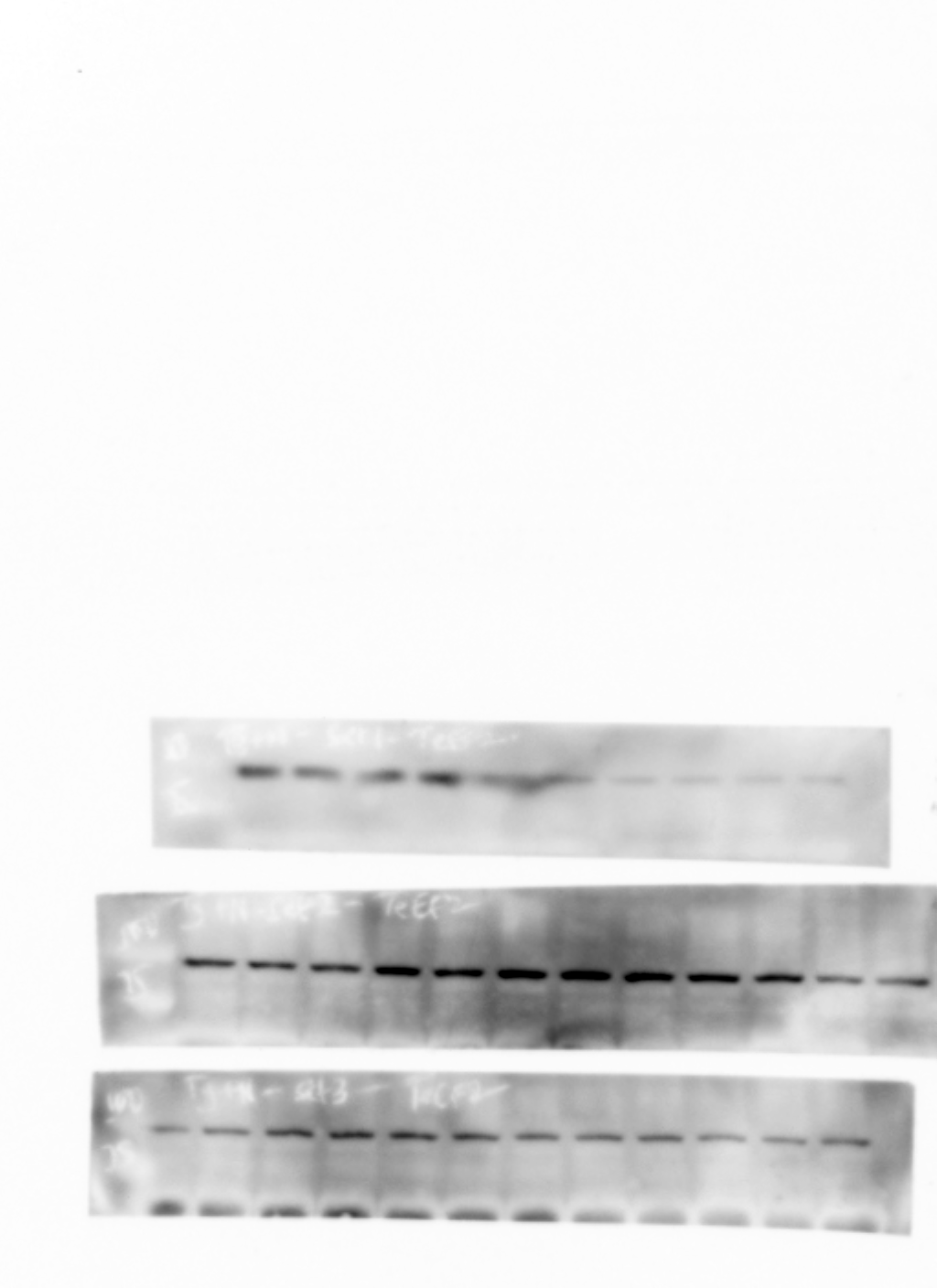
**

**Phospho eEF2:**

Loading order for Phospho - ladder, test sample (it was just an extra sample i had loaded), n1', n5', n20', unt, tg 5', tg 20', tg+n1', tg+n20'

**
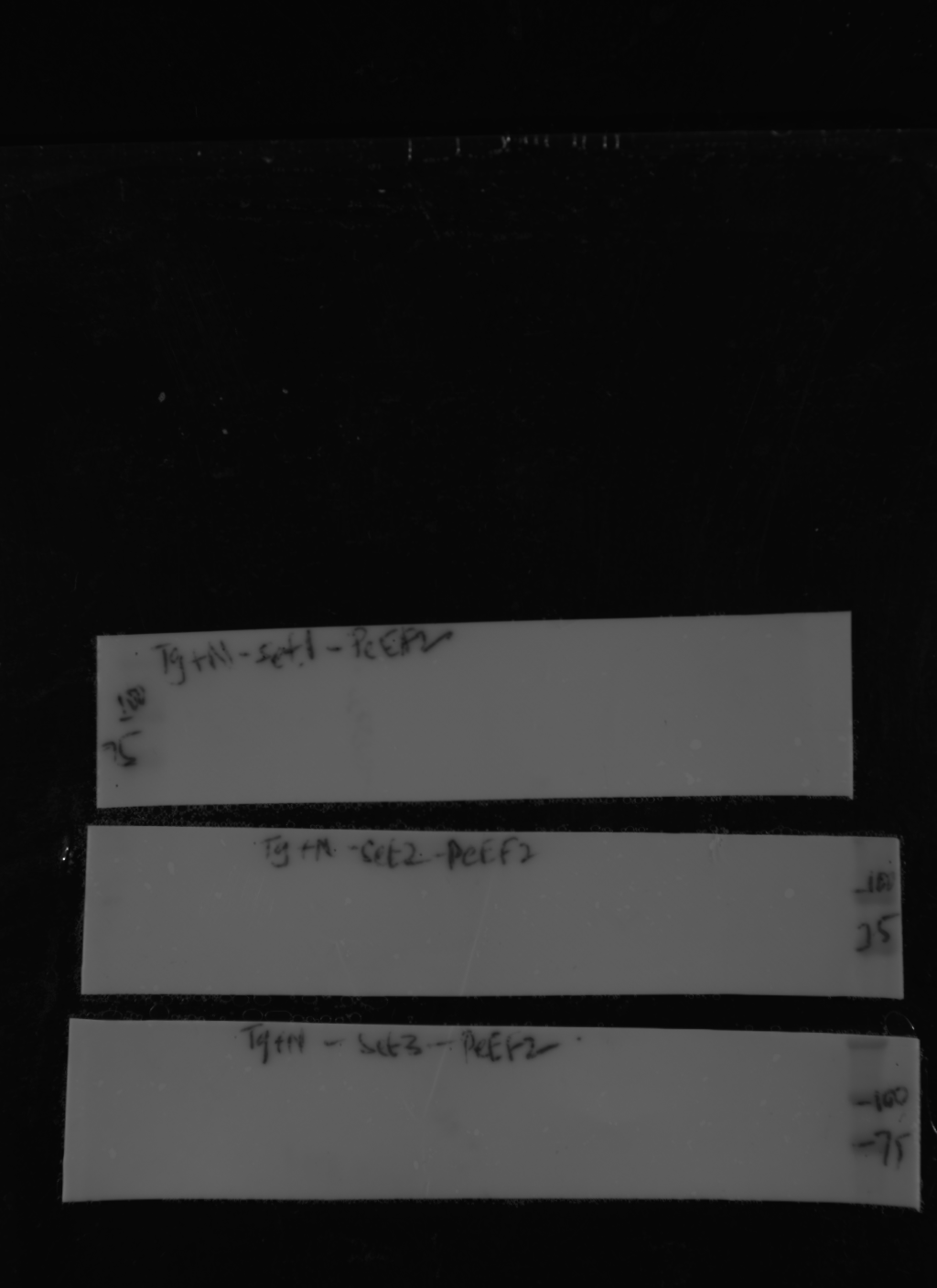
** **
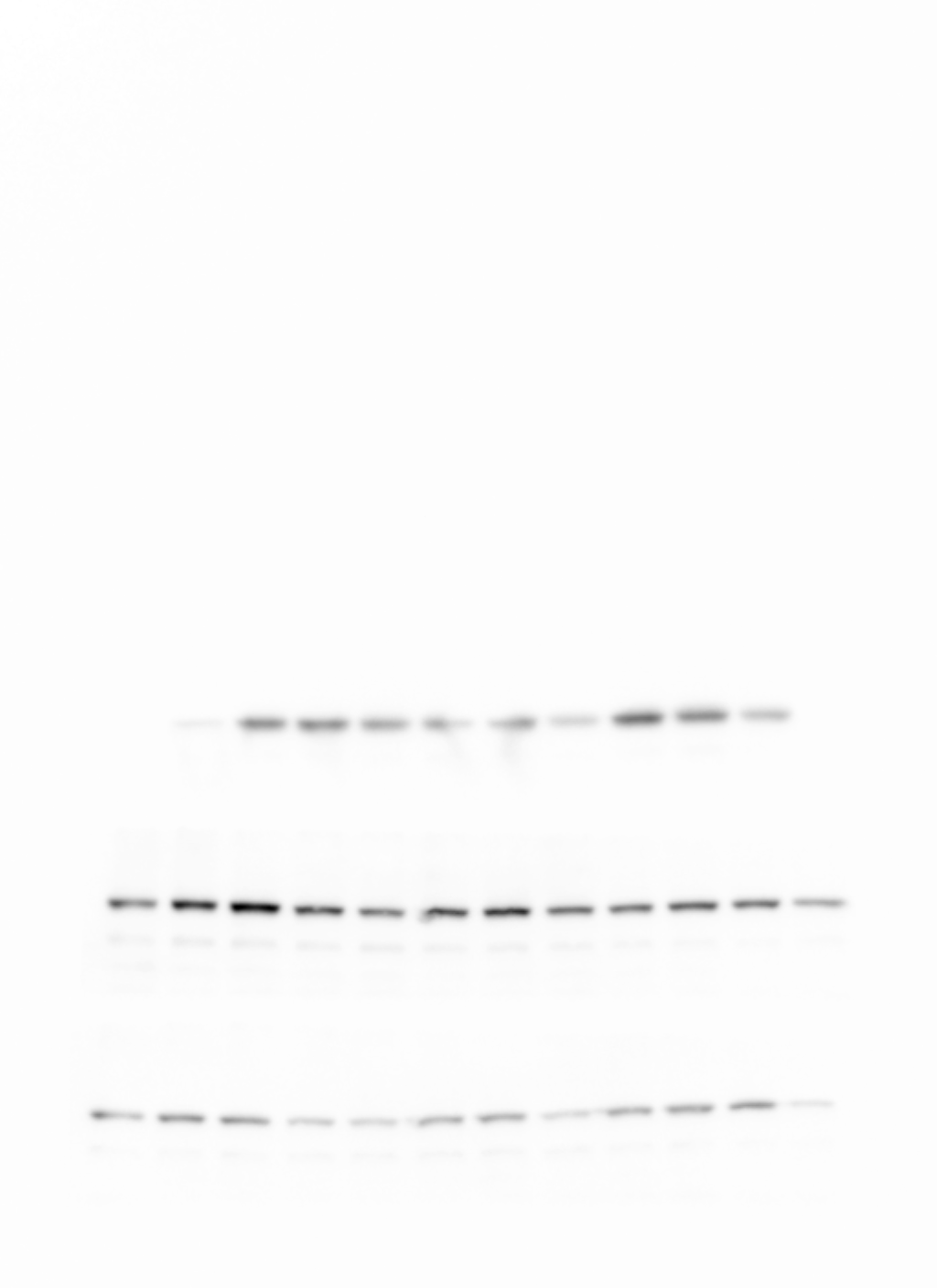
**

**Tuj1:**

**First two blots are for the p-eEF2 and eEF2**

**
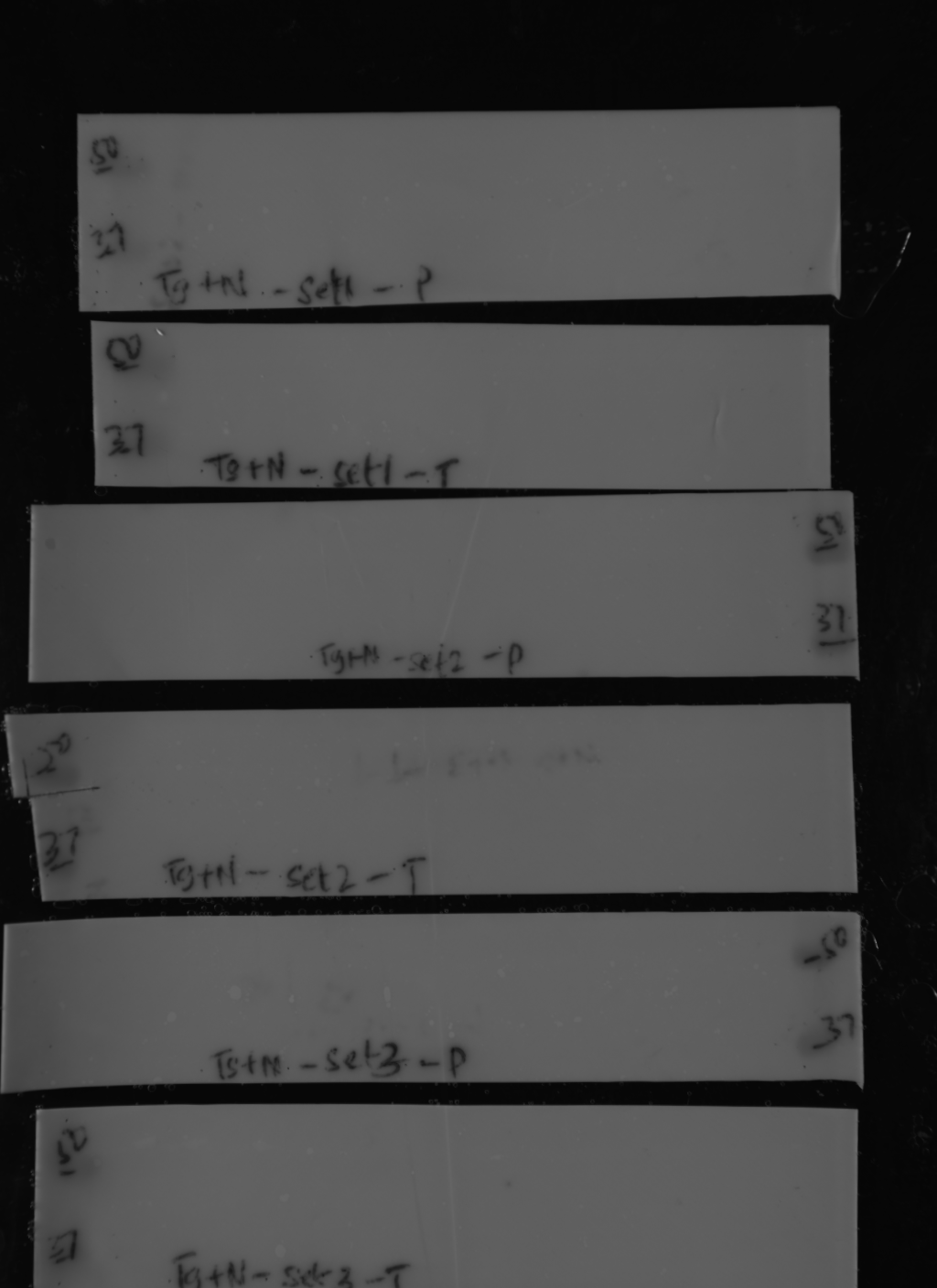
** **
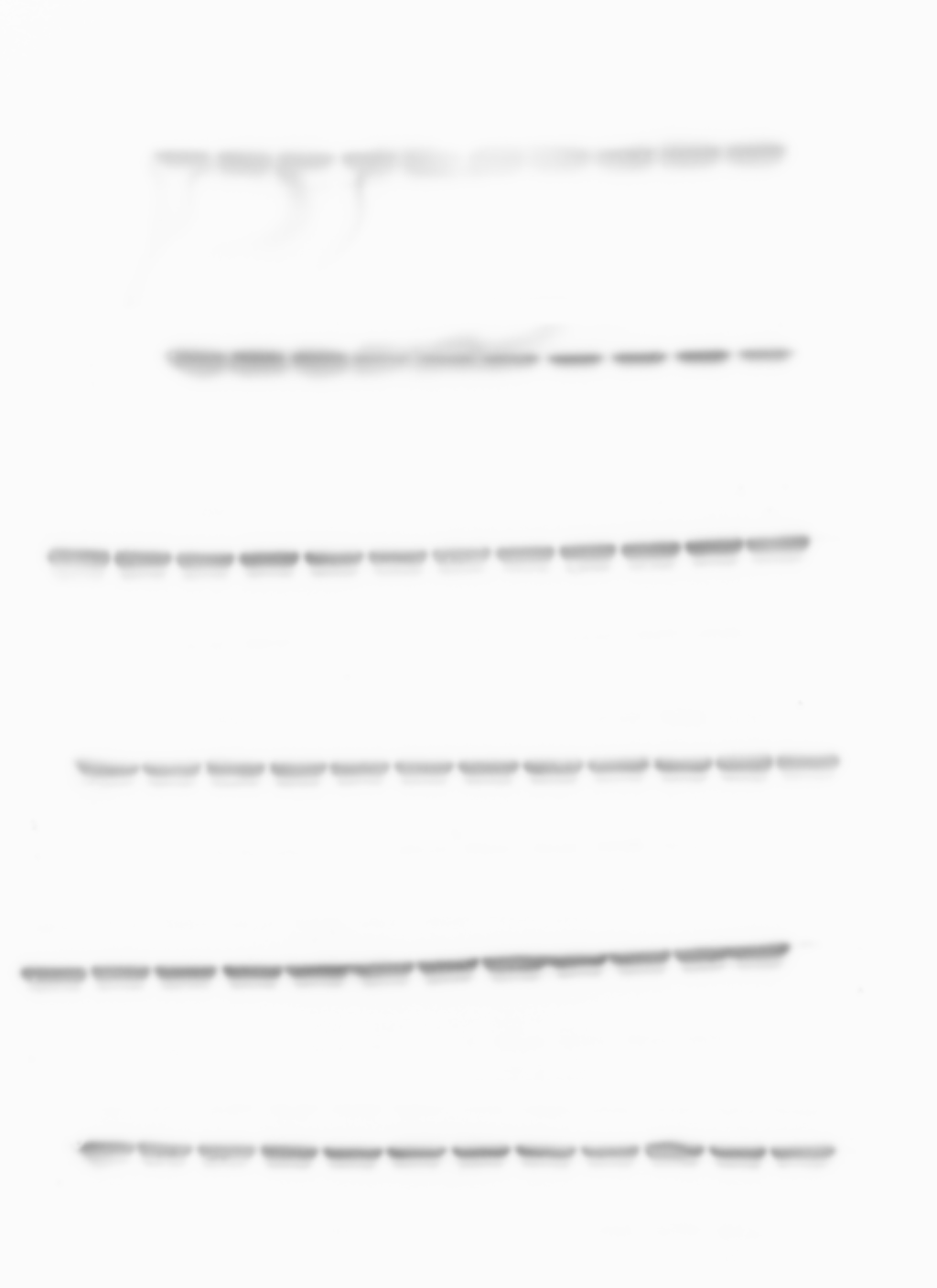
**

**Set4: The NMDA treated samples are loaded along with Calcium treatment- for quantification we have used Set1 samples.**

**Total eEF2:** Loading order for total S1 - ladder, unt, n1', n5', n20', unt-ca, n1'-ca, n5'-ca, n20-ca

**
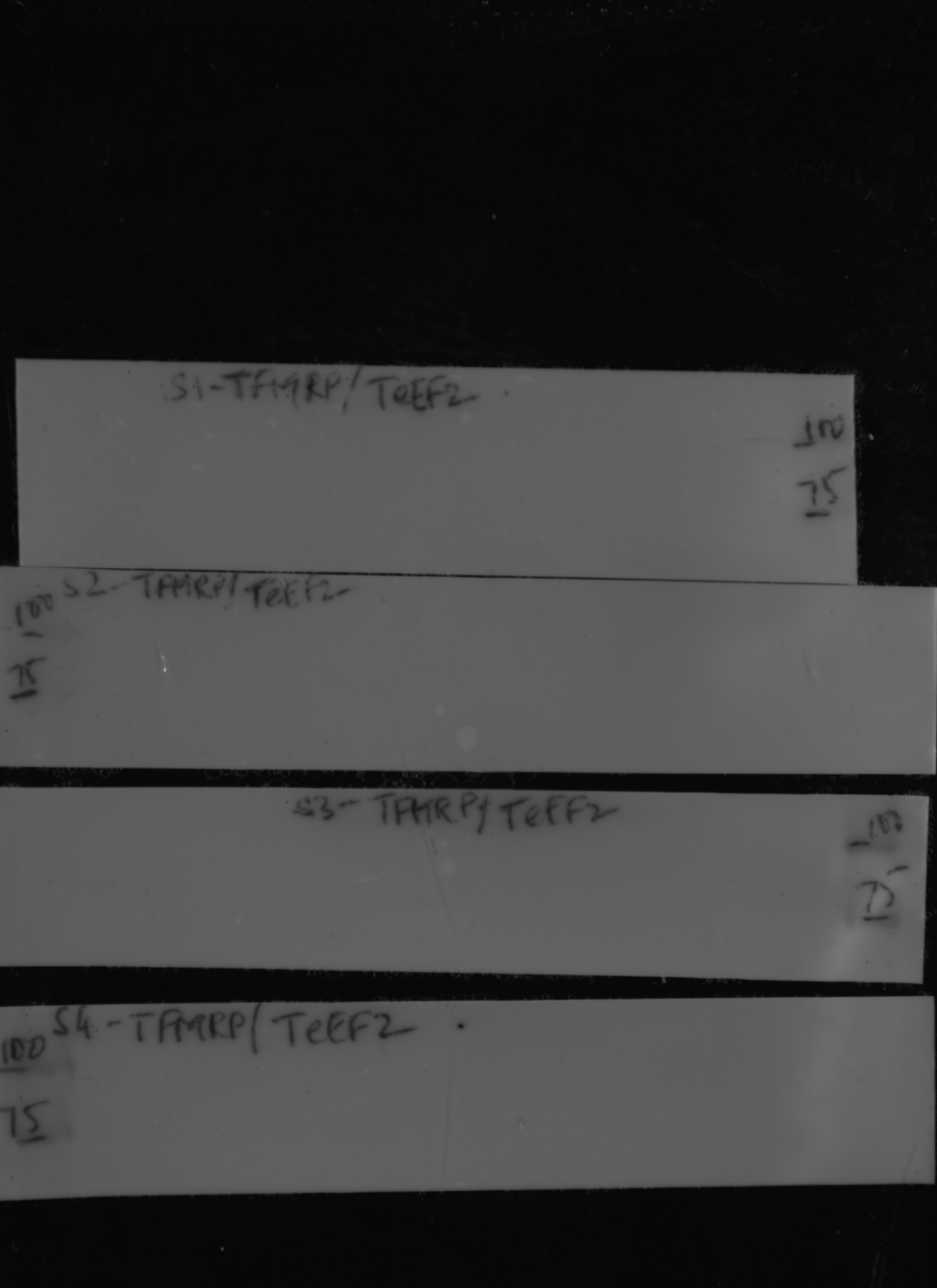
** **
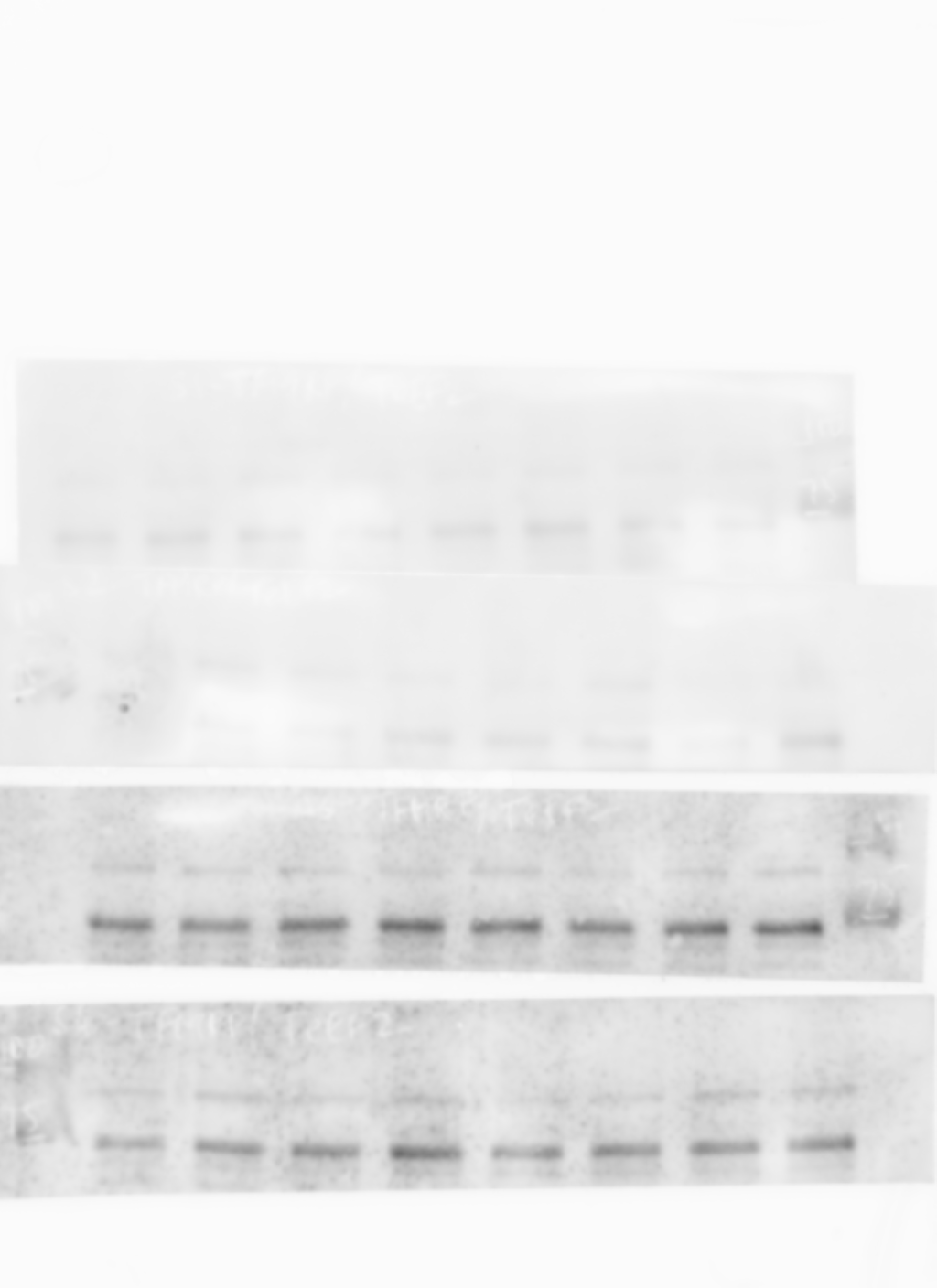
**

**Phospho eEF2:**

Loading order for phosphor S1 - ladder, unt, n1', n5', n20', unt-ca, n1'-ca, n5'-ca, n20-ca

**
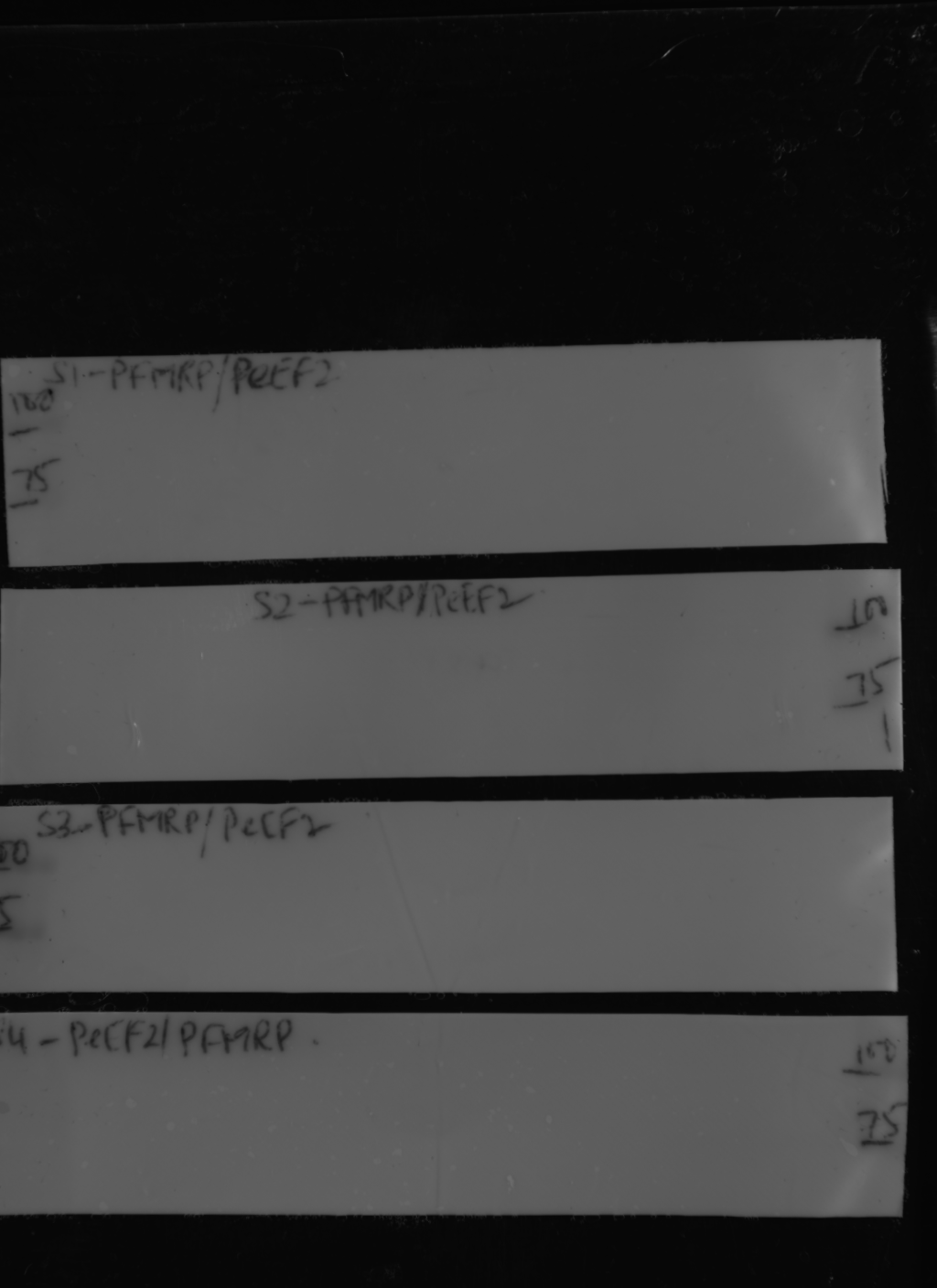
** **
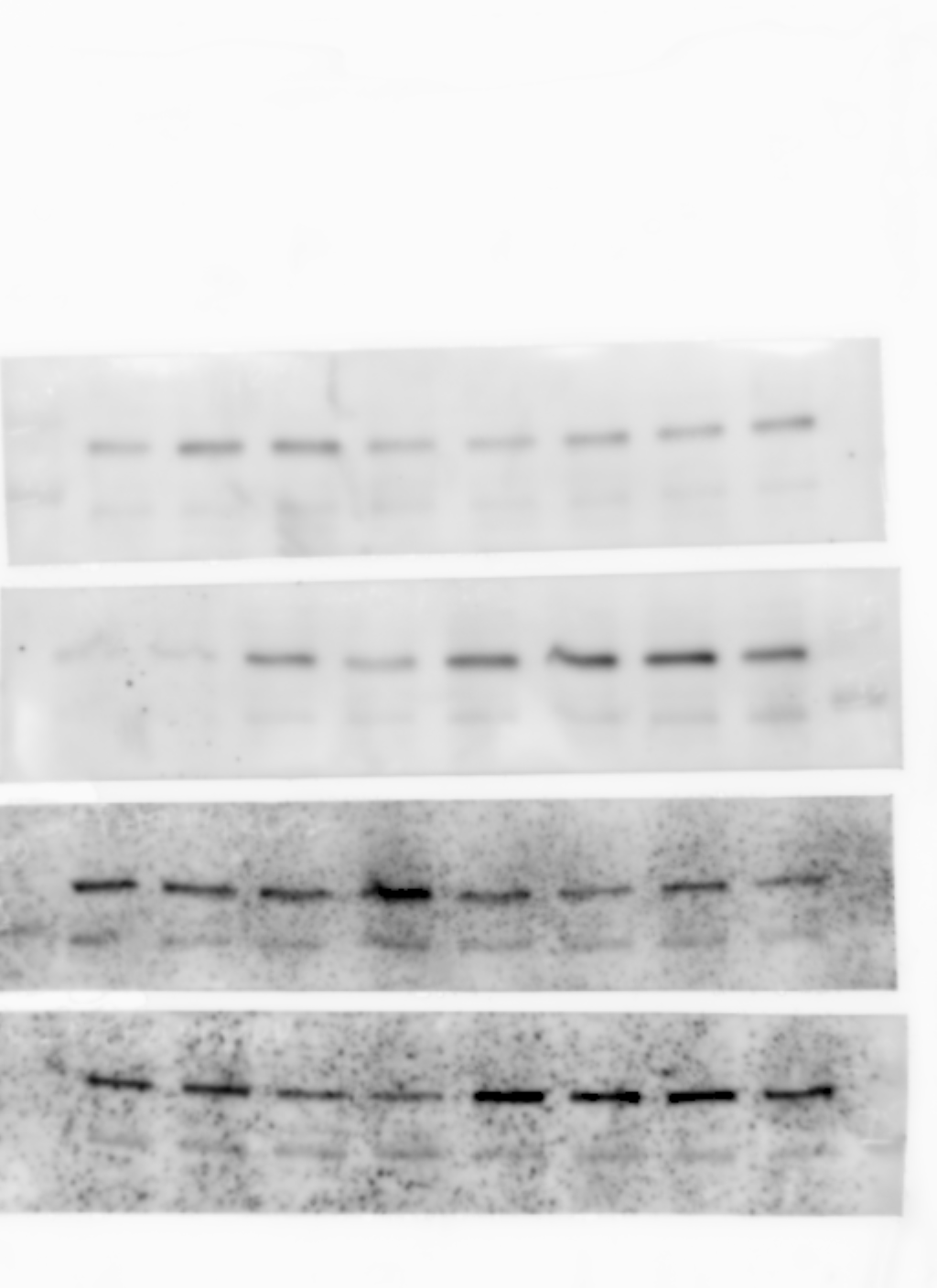
**

**Tuj1: First two blots for Phospho and total eEF2**

**
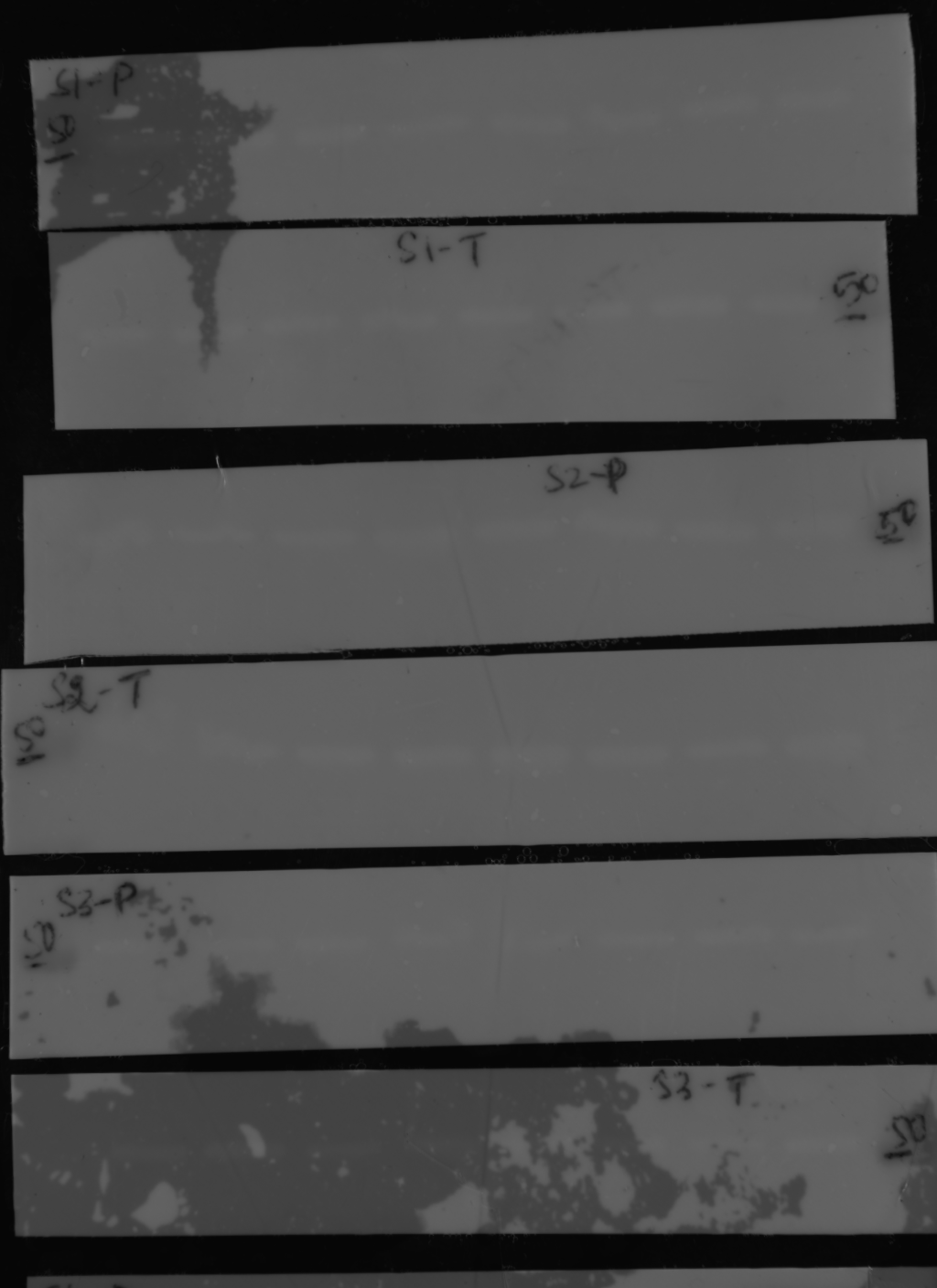
** **
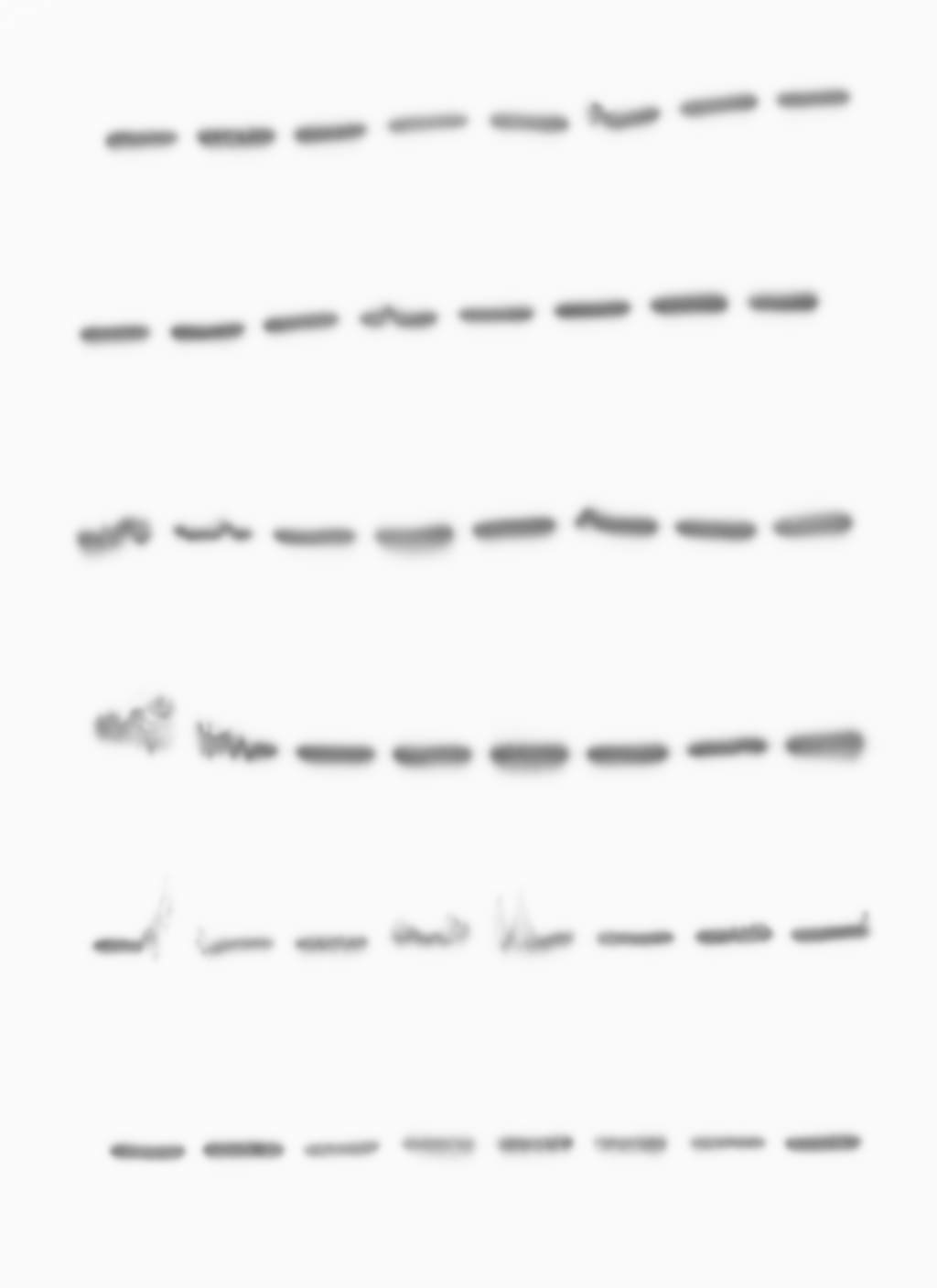
**

**Figure 2: NMDA induces changes in nuclear and cytosolic levels of m^6^A demethylase FTO**

**E:**

**FTO western blot – Red box indicate the representative image**

**Set1 and 2:**

**FTO Loading order : Right to left 🡪Basal – NMDA 1’ – NMDA 5’ – NMAD 20’**

**
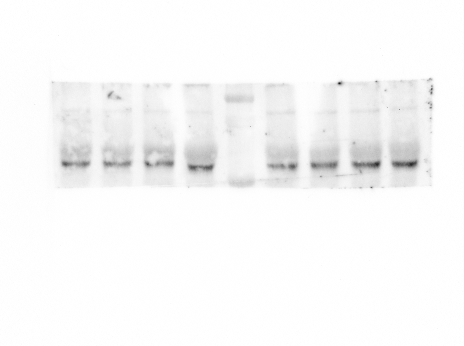
**

**GAPDH Western blot :**

**GAPDH Loading order : Right to Left 🡪 Basal – NMDA 1’ – NMDA 5’ – NMAD 20’**

**
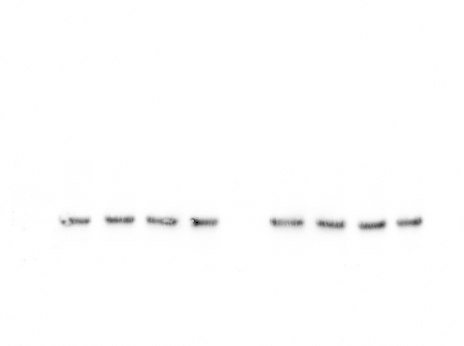
**

**Set 5 and 6:**

**FTO**

**FTO Loading order : Left to right 🡪Basal – NMDA 1’ – NMDA 5’ – NMAD 20’**

**
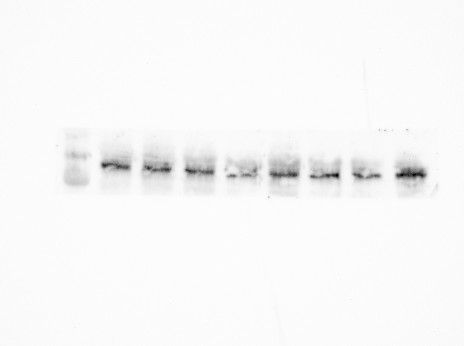
**

**GAPDH:**

**GAPDH Loading order : Left to Right 🡪 Basal – NMDA 1’ – NMDA 5’ – NMAD 20’**

**
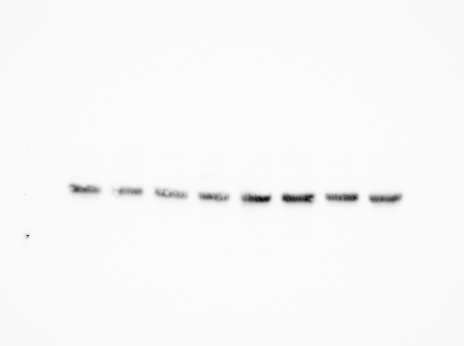
**

**Figure 3: NMDAR mediated increase in m6A levels is accompanied with shift of m6A marked RNA from polysome to non-polysome fractions**

**C:**

**
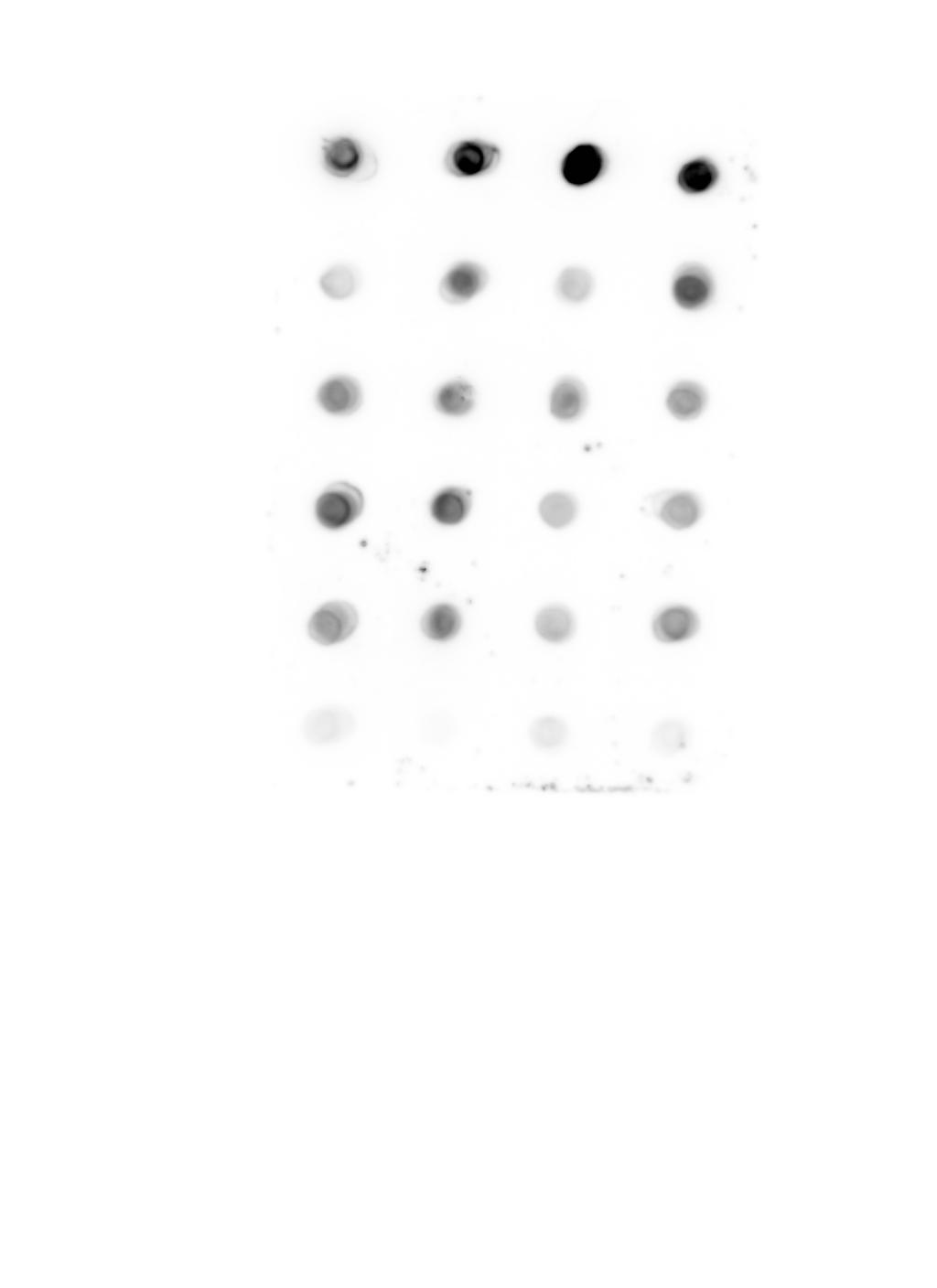
**

**
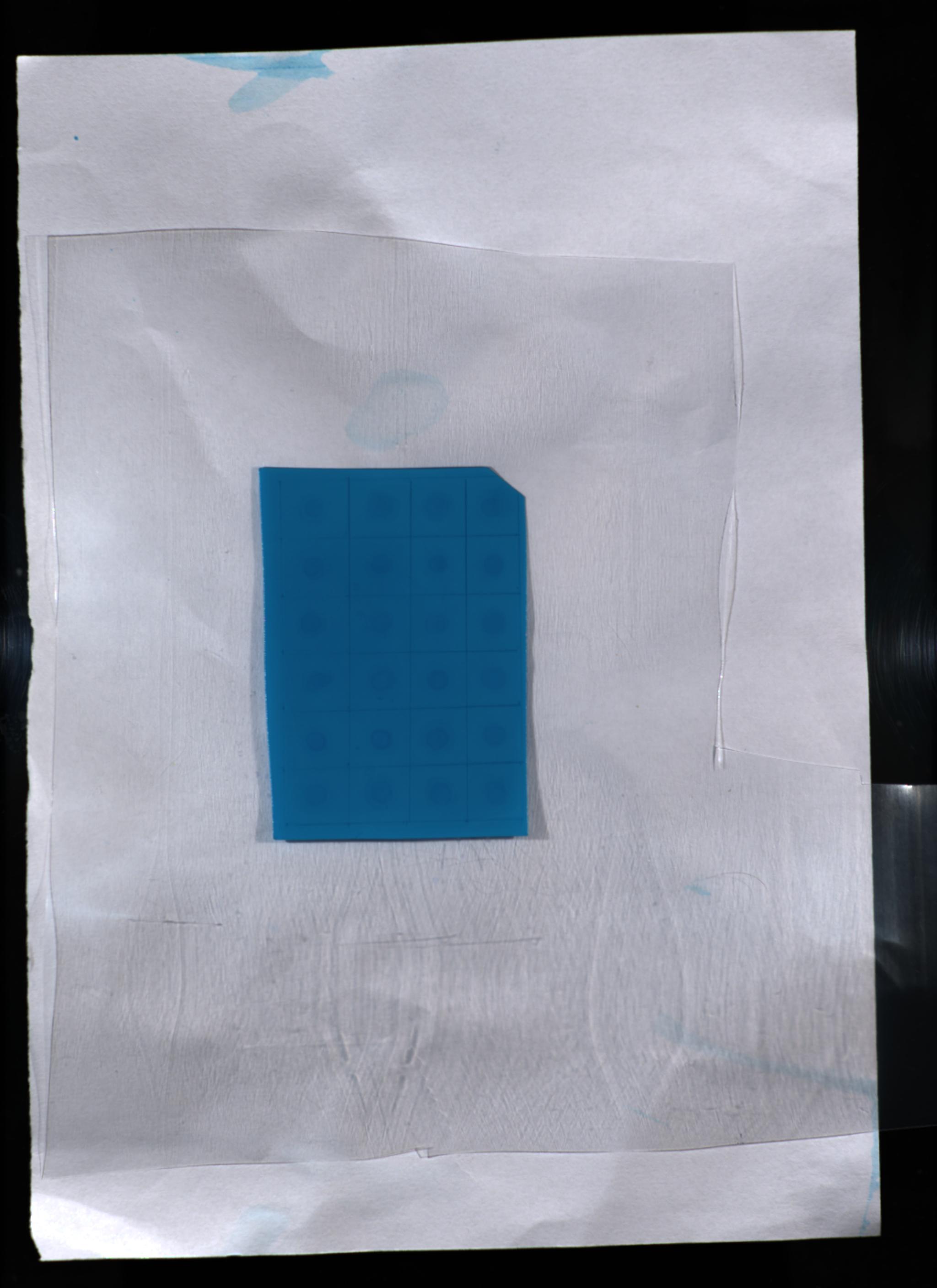
**
